# Eicosapentaenoic acid increases proportion of type 1 muscle fibers through PPARδ and AMPK pathways in rats

**DOI:** 10.1016/j.isci.2024.109816

**Published:** 2024-04-26

**Authors:** Yusuke Komiya, Yuka Sakazaki, Tsuyoshi Goto, Fuminori Kawabata, Takahiro Suzuki, Yusuke Sato, Shoko Sawano, Mako Nakamura, Ryuichi Tatsumi, Yoshihide Ikeuchi, Keizo Arihara, Wataru Mizunoya

**Affiliations:** 1 Department of Animal Science, School of Veterinary Medicine, Kitasato University, Towada, Japan; 2 Department of Animal and Marine Bioresource Sciences, Faculty of Agriculture, Graduate School of Agriculture, Kyushu University, Fukuoka, Japan; 3 Division of Food Science & Biotechnology, Kyoto University, Kyoto, Japan; 4 Faculty of Agriculture and Life Science, Hirosaki University, Hirosaki, Japan; 5 Department of Animal Science, School of Agriculture, Tokai University, Kumamoto, Japan; 6 Department of Food and Life Science, School of Life and Environmental Science, Azabu University, Sagamihara, Japan; 7 Department of Animal Science and Biotechnology, School of Veterinary Medicine, Azabu University, Sagamihara, Japan

**Keywords:** Dietary supplement, Molecular biology, Metabolomics, Transcriptomics

## Abstract

Muscle fiber type composition (% slow-twitch and % fast-twitch fibers) is associated with metabolism, with increased slow-twitch fibers alleviating metabolic disorders. Previously, we reported that dietary fish oil intake induced a muscle fiber-type transition in a slower direction in rats. The aim of this study was to determine the functionality of eicosapentaenoic acid (EPA), a unique fatty acid in fish oil, to skeletal muscle fiber type and metabolism in rats. Here, we showed that dietary EPA promotes whole-body oxidative metabolism and improves muscle function by increasing proportion of slow-twitch type 1 fibers in rats. Transcriptomic and metabolomic analyses revealed that EPA supplementation activated the peroxisome proliferator-activated receptor δ (PPARδ) and AMP-activated protein kinase (AMPK) pathways in L6 myotube cultures, which potentially increasing slow-twitch fiber share. This highlights the role of EPA as an exercise-mimetic dietary component that improves metabolism and muscle function, with potential benefits for health and athletic performance.

## Highlights

• Eicosapentaenoic acid (EPA) possesses high PPARδ-agonist activity
• EPA intake increases proportion of slow-twitch type 1 muscle fibers
• EPA intake promotes oxidative metabolism and improves muscle function
• EPA activates both PPARδ and AMPK pathways

## Graphical abstract

**Figure:**
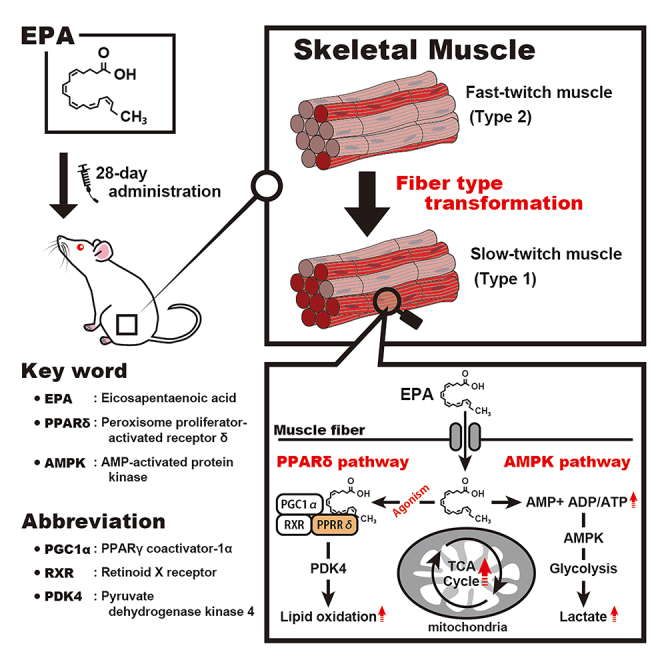

## Introduction

Chronic physical inactivity, such as long-term desk-based work, induces perturbations in skeletal muscle metabolism, negatively impacts the quality of life, and increases the risks for various metabolic diseases, such as type-2 diabetes and cardiovascular diseases,^1^ which are serious public health and economic problems. Furthermore, the increased prevalence of remote work owing to the coronavirus disease 2019 pandemic is expected to negatively impact physical inactivity,^2^^,^^3^ and we believe that the improvement of muscle metabolism is key for solving related problems.

Contractile function and high energy consumption are two important skeletal muscle characteristics. These characteristics depend on the composition of the skeletal muscle fiber type. Two major types of muscle fibers are found in mammalian skeletal muscles, namely type 1 (slow-twitch and oxidative; red muscles) and type 2 fibers (fast-twitch and glycolytic; white muscles), which are subdivided into types 2A, 2X, and 2B in order of increasing fast-twitch fiber’s traits.^4^^,^^5^ Type 1 fibers contain more mitochondria, possess high oxidative capacity, and are resistant to fatigue. In contrast, type 2 muscle fibers undergo high rates of glycolytic metabolism and show fatigue. Thus, muscles enriched in type 1 fibers typically perform sustained and tonic contractile activities such as postural tension, whereas muscles enriched in type 2 fibers are typically involved in intense and rapid activities over a short duration. Therefore, the composition of muscle fiber types determines various contractile and metabolic properties of skeletal muscle, including high-speed muscle strength and endurance performance. Exercise improves muscle properties, such as contraction, metabolism, and function.^6^

Several studies have shown that in addition to exercise, various dietary components can alter muscle characteristics; such foods are termed as “exercise mimetics.” Previously, we found that dietary fish oil intake induced the muscle fiber-type transition to a slower direction (type 2B-to-2X transition) and increased the levels of oxidative metabolism-related factors in the extensor digitorum longus (EDL) muscles of rats.^7^ We observed accumulation of the fish oil-specific fatty acids eicosapentaenoic acid (EPA, 20:5) and docosahexaenoic acid (DHA, 22:6) in the muscles of fish oil-fed rats. EPA and DHA are essential fatty acids classified as *n*-3 polyunsaturated fatty acids (PUFA) and are known as bioactive compounds, reported in numerous studies. For example, EPA and DHA increase hepatic fatty acid oxidation and reduce triacylglycerol (TAG) synthesis by downregulating the expressions of sterol regulatory element-binding protein-1 and carbohydrate response element-binding protein which activate hepatic lipogenesis.^8^ In adipose tissue, they decrease the release of fatty acids and adipokines and have an anti-inflammatory effect by inhibiting recruitment and activation of macrophages,^9^ and they induce UCP1 expression via sympathetic nervous system activation.^10^ On the other hand, EPA and DHA act independently via different mechanisms although they are very similar in structure and share some metabolic effects. A highly purified EPA ethyl ester significantly reduced the risk of cardiovascular death, myocardial infarction in patients with hypertriglyceridemia.^11^ EPA supplementation also reduced the serum levels of small-dense low-density lipoprotein^12^ and improved TAG metabolism in obesity.^13^

EPA and DHA also act as bioactive compounds in skeletal muscle. EPA increases muscle protein synthesis with activating of mTORC1 signal and attenuating the rate of protein degradation in C2C12 myotubes.^14^ DHA prevents palmitate-induced activation of proteolysis pathways, such as ubiquitin-proteasome and autophagy-lysosome systems, in C2C12 myotubes,^15^ and improves mitochondrial enzyme activities and increased oxygen consumption in soleus muscle of adult rats.^16^ However, the effect of EPA and DHA on muscle fiber type has not been elucidated.

One of the important target of EPA and DHA in skeletal muscle cells is peroxisome proliferator-activated receptor (PPAR)δ which is a nuclear receptor belonging to the PPAR family. EPA and DHA are ligands for PPARδ.^17^ Overexpression of the constitutively activated form of PPARδ upregulated various factors related to oxidative metabolism and increased type 1 fibers in mice.^18^ Oral administration of the PPARδ-specific agonist GW501516 induced an increase in type 1 fibers in mice^18^^,^^19^ and treatment of GW501516 increased mitochondrial content and function in C2C12 myotubes.^20^

These reports led us to hypothesize that fish oil-specific *n*-3 PUFA activates PPARδ in skeletal muscles and contributes to muscle fiber-type transitions. To test this hypothesis, we investigated the effects of *n*-3 PUFA, especially EPA, on skeletal muscle fiber types *in vivo* and *in vitro* by focusing on PPARδ activation, muscle contractile, and metabolic characteristics.

## Results

### EPA, not DHA, activates PPARδ *in vitro*

**Figure 1:**
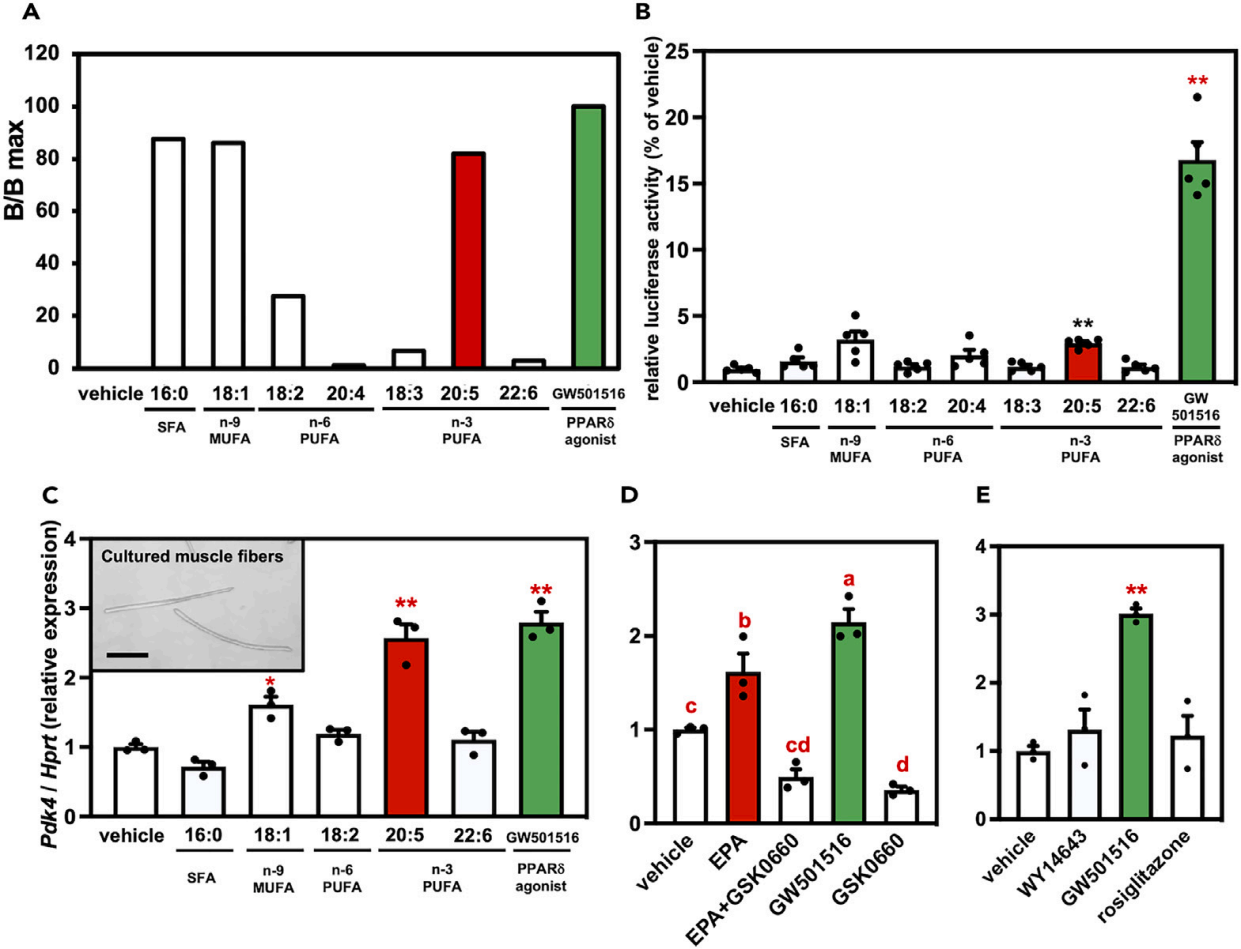
Measurement of PPARδ-agonist activities of fatty acids including EPA *in vitro* (A) The peroxisome proliferator-activated receptor (PPAR)δ-agonist activity of fatty acids measured by a nuclear receptor cofactor assay system. The intensity of the GW501516 treatment was set at 100% (B/Bmax), and relative intensity is presented as the fold induction relative to that of the GW501516. (B) Effects of fatty acids on the activation of PPARδ in a luciferase reporter assay system using GAL4/PPAR chimera proteins. The cells were treated with 30 μM fatty acids for 16 h. The activity of the vehicle control was set at 100%, and relative luciferase activity is presented as the fold induction relative to that of the vehicle control. Data are means ± SEM of five tests (∗∗*p* < 0.01 compared with the vehicle controls). (C) Effects of fatty acids on the transcript expression of *Pdk4* genes in muscle fibers isolated from flexor digitorum brevis (FDB) muscle. The image embedded in the graph shows cultured muscle fibers isolated from FDB muscle. The bars indicate 200 μm. The values are shown as the means SEM (*n* = 3 independent cultures, ∗∗*p* < 0.01 and ∗*p* < 0.05 compared with the vehicle controls). (D) Effects of eicosapentaenoic acid (EPA) with GSK0660, a PPARδ antagonist, on the transcript expression of *Pdk4* genes in muscle fibers isolated from FDB muscle. Data are means ± SEM (*n* = 3 independent cultures, different superscripts indicate a significant difference between two groups). (E) Effects of PPAR agonists on the transcript expression of *Pdk4* genes in muscle fibers isolated from FDB muscle. WY14643, GW501516, and rosiglitazonee were used for PPARα, PPARδ, and PPARγ, respectively. Data are means ± SEM (*n* = 3 independent cultures, ∗∗*p* < 0.01 compared with the vehicle controls).

To confirm the type of fatty acids involved in the activation of PPARδ, we applied three different assays to measure the PPARδ-agonist activities of several fatty acids, including EPA and DHA, *in vitro*. First, we performed a coactivator-binding assay and found that EPA, palmitic acid, and oleic acid possessed high PPARδ-agonist activities (Figure 1A). We next analyzed PPARδ-agonist activities in a luciferase reporter assay with the CV-1 monkey kidney cell line.^21^ We found that cells supplemented with EPA showed significantly high luciferase activities (*p* < 0.01, Figure 1B). EPA showed potent PPARδ-agonistic activity in both assays. To determine whether PPARδ activation could be induced in muscle cells, we utilized isolated rat muscle fibers exposed to fatty acids and measured the mRNA levels of *Pdk4*, a representative PPARδ target gene.^22^ Supplementation with EPA (*p* < 0.01) and oleic acid (*p* < 0.05) significantly increased *Pdk4* expression in isolated muscle fibers (Figure 1C). Furthermore, to clarify whether increased *Pdk4* expression could be induced through PPARδ activation, muscle fibers were treated with EPA in the presence of the PPARδ antagonist GSK0660. The results showed that *Pdk4* upregulation by EPA supplementation was significantly suppressed by co-supplementation with GSK0660 (*p* < 0.01) (Figure 1D). We further examined the contribution of other PPAR isoforms (PPARα and PPARγ).^23^ We analyzed *Pdk4* expression in muscle fibers treated with the PPAR isoform-specific agonists WY14643 (PPARα), GW501516 (PPARδ), and rosiglitazone (PPARγ). Only supplementation with GW501516 significantly upregulated *Pdk4* expression in muscle fibers (*p* < 0.01) (Figure 1E), suggesting that PPARδ is the principal isoform in skeletal muscles. Interestingly, DHA, another fatty acid present in fish oil, exhibited little PPARδ-agonist activity in any of the assays. Therefore, we hypothesized that EPA activates PPARδ in skeletal muscles.

### EPA upregulates the mRNA and protein expression levels of oxidative metabolic genes in muscle cells

**Figure 2:**
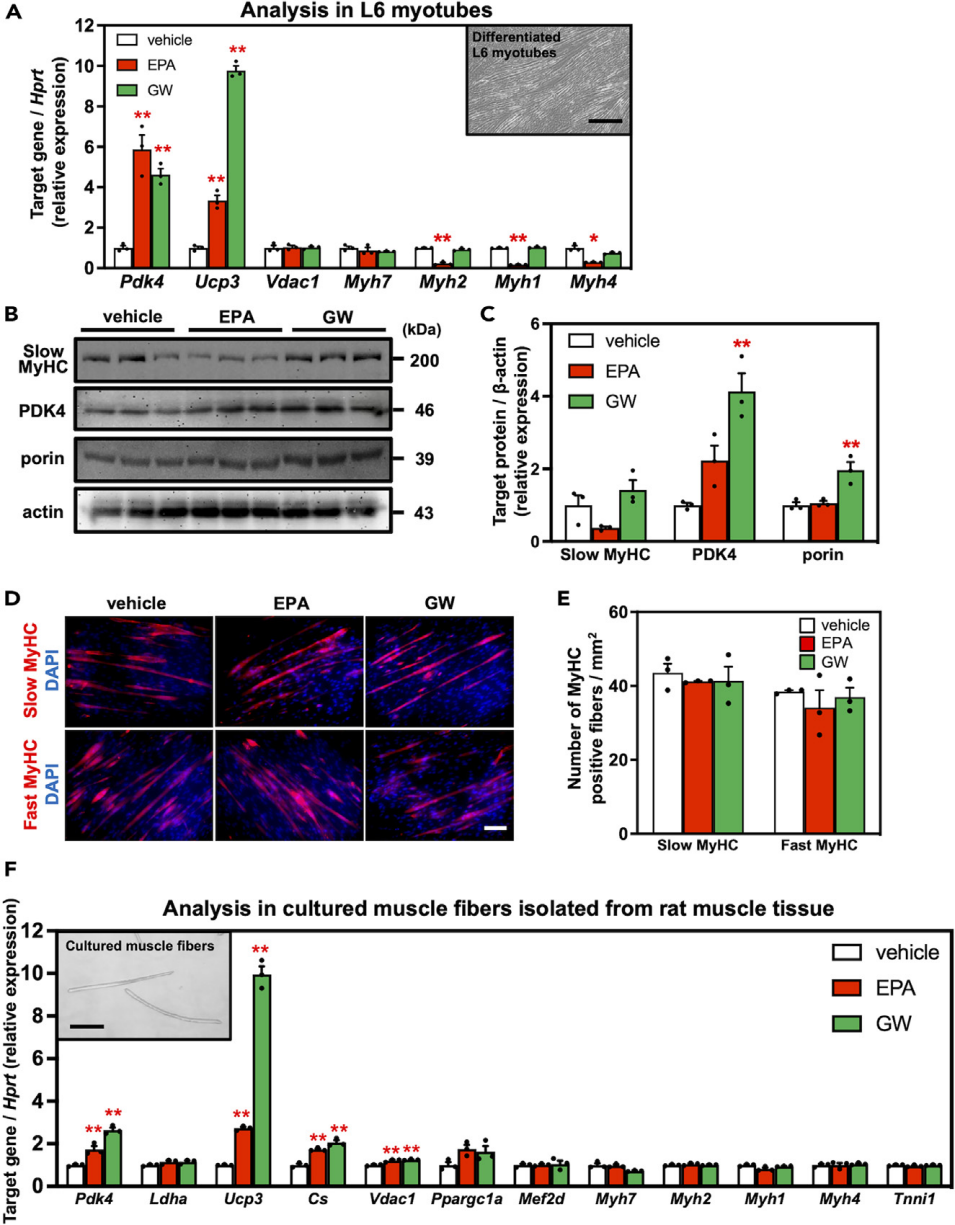
Effect of EPA supplementation on mRNA and protein expression of muscle fiber type related factors in muscle cells The mRNA (A) and protein (B and C) expression in differentiated L6 myotubes supplemented with eicosapentaenoic acid (EPA). The image embedded in the graph (A) shows differentiated L6 myotubes. The bars indicate 200 μm. Cells were treated with 30 μM EPA or 100 nM GW501516 for 12 h (mRNA) or 120 h (protein). (D) Immunofluorescence microscopy for slow/fast MyHC (red) and DAPI (blue) shows fast-/slow-twitch myotubes and nuclei, respectively. Cells were treated with 30 μM EPA or 100 nM GW501516 for 120 h.The scale bar indicates 100 μm. (E) The number of each MyHC positive fiber was standardized to the area. (F) The mRNA expression in muscle fibers isolated from flexor digitorum brevis (FDB) muscle supplemented with EPA. The image embedded in the graph shows cultured muscle fibers isolated from FDB muscle. The bars indicate 200 μm. Cells were treated with 30 μM of EPA or 100 nM GW501516 for 12 h. Data are means ± SEM (*n* = 3 independent cultures, ∗∗*p* < 0.01 and ∗*p* < 0.05 compared with the vehicle controls).

We next investigated the effects of EPA on the mRNA and protein expression levels of several key genes related to muscle fiber types and oxidative metabolism in differentiated L6 myotube cultures. Treating myotubes with EPA for 12 h resulted in a significant increase in the mRNA levels of *Pdk4* (*p* < 0.01) and *Ucp3* (*p* < 0.01), and similar changes were observed after GW501516 stimulation. The mRNA expression of *Myh7*, a marker of type 1 fibers, did not change after each treatment, whereas the mRNA expression of *Myh2* (*p* < 0.01), *Myh1* (*p* < 0.01), and *Myh4* (*p* < 0.05) was significantly lower in the EPA group (Figure 2A), which is a marker of type 2A, 2X, and 2B fibers, respectively. The changes in *Myh* expression were observed after EPA treatment, but not after GW501516 treatment, suggesting the existence of PPARδ-independent regulation. The protein expression of slow MyHC did not change in both EPA and GW groups (Figures 2B and 2C). We examined the composition of muscle fiber types in the myotubes by immunocytochemistry; however, no changes in either slow or fast MyHC were observed with EPA and GW treatment (Figures 2D and 2E). PDK4 and porin expressions were upregulated at the protein level by GW501516 treatments (Figures 2B and 2C).

Previously, we reported that cultured myotubes are immature and different from mature muscle fibers in terms of the expression levels of contractile proteins associated with muscle fiber types as well as their cell morphology, suggesting that isolated single muscle fibers are a superior *in vitro* model for studying muscle cells in their native form.^24^^,^^25^ Thus, we supplemented the culture medium for isolated muscle fibers with EPA or GW501516, and found that EPA supplementation significantly increased the mRNA levels of genes related to oxidative metabolism (*Pdk4*, *Ucp3*, and *Cs*) and mitochondria (*Vdac1*), whereas the expression levels of genes associated with contractile properties (*Myh1/2/4/7* and *Tnni1*) were not altered in isolated fibers, and most upregulated genes were consistent with those in the GW group (Figure 2F). The data suggest that EPA treatment upregulated key genes involved in oxidative metabolism via PPARδ activation in both myotubes and muscle fibers *in vitro*. However, alterations in the expression levels of genes associated with contractile properties were limited to L6 myotubes.

### EPA increases whole-body fat oxidation and muscle performance in rats

**Figure 3:**
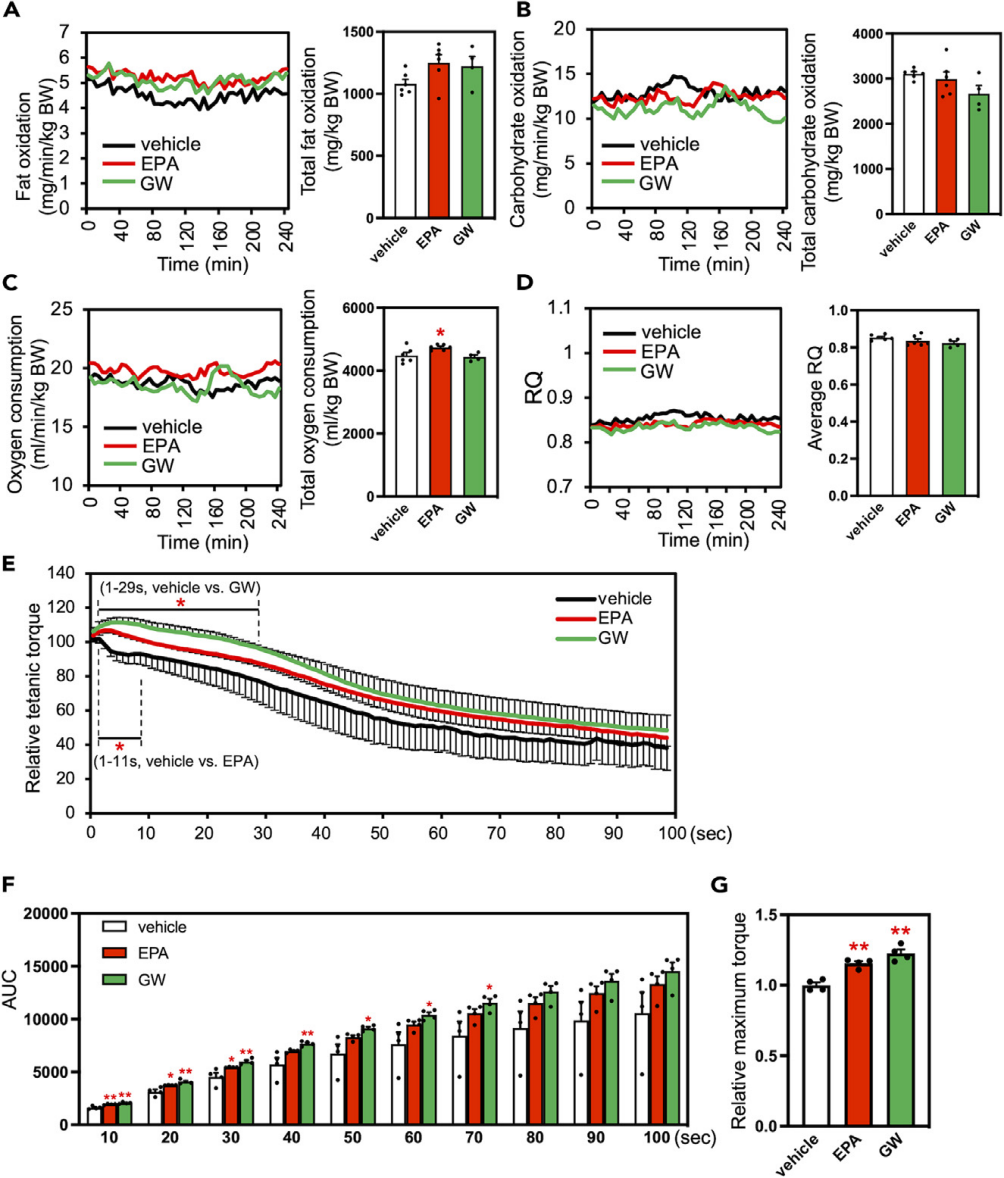
Effect of 4-week EPA supplementation on systemic metabolism and muscle performance in rats Time-course changes and calculated fat oxidation (A), carbohydrate oxidation (B), oxygen consumption (C), and respiratory quotient (RQ; D) of rats administrated with EPA (1000 mg/kg/day) or GW501516 (10 mg/kg/day) for 4 weeks. Data are means ± SEM (*n* = 6 in vehicle and EPA group, *n* = 4 in GW501516 group, ∗*p* < 0.05 compared with the vehicle controls). The time-course change in response to 100-s successive stimulation (E, expressed relative to the initial value). Area under the curve (AUC) and relative maximum torque from tetanic-contraction are shown in (F and G), respectively. Data are means ± SEM (*n* = 4, ∗∗*p* < 0.01 and ∗*p* < 0.05 compared with the vehicle controls).

Since we found that EPA activated PPARδ and increased the mRNA levels of oxidative metabolic genes in muscle cells *in vitro*, we tested whether EPA could activate PPARδ *in vivo*. Rats were orally administered 1000 mg/kg EPA ethyl ester or 10 mg/kg GW501516 daily for 4 weeks. After this period, we examined systemic metabolic alterations using respiratory gas analysis. The results were largely similar between the EPA and GW treatment. Fat oxidation (*p* = 0.089, Figure 3A) and oxygen consumption (*p* < 0.05, Figure 3C) were higher in the EPA group than in the control group. Carbohydrate oxidation (*p* = 0.082, Figure 3B) and the respiratory quotient (RQ; *p* = 0.098, Figure 3D) were lower in the GW group than in the control group. Although detected differences were different between the EPA and GW groups, these results are commonly interpreted as a shift of consumed energy substrate from carbohydrates to fatty acids by chronic EPA or GW administration. In our pilot study, we examined the respiratory gas analysis during 24-h period covering light and dark periods to confirm the circadian alterations, and we found fat oxidation was increased and RQ was decreased in the EPA administered group (Figures S1A and S1D) in agreement with our 4-h gas analysis although there were some limitations in this pilot study (see the legend of Figure S1).

Then, we investigated whether a 4-week administration of EPA or GW501516 led to the functional impact of changes in rat muscle performance. We measured successive maximum isometric plantar-flexion force conducted by electrical tibial nerve stimulation under anesthesia. The results showed that the decline in tetanic muscle force was attenuated in the EPA group and GW group than in the control group (Figure 3E). To quantify the muscle endurance, we analyzed area under the curve (AUC) from tetanic muscle force. The AUC was significantly higher in the EPA group (until 30 s) and GW group (until 70 s) than in the control group (Figure 3F). Because the increase of AUC (attenuated decline in muscle force) indicates improved tolerance to exhaustion, it is suggested that EPA and GW treatment increased muscle endurance. Moreover, the relative maximum torque, which indicates the potential muscle strength, was higher in the EPA and GW groups than in the control group (*p* < 0.01, Figure 3G). These data suggest that EPA administration improved whole oxidative metabolism and muscle function in rats. The harvested skeletal muscle tissue weights were not significantly different among these groups (Table S1), suggesting such improvement in muscle functions is not due to skeletal muscle mass alteration.

### EPA upregulates slow-MyHC expression in rat muscles after a 4-week administration

**Figure 4:**
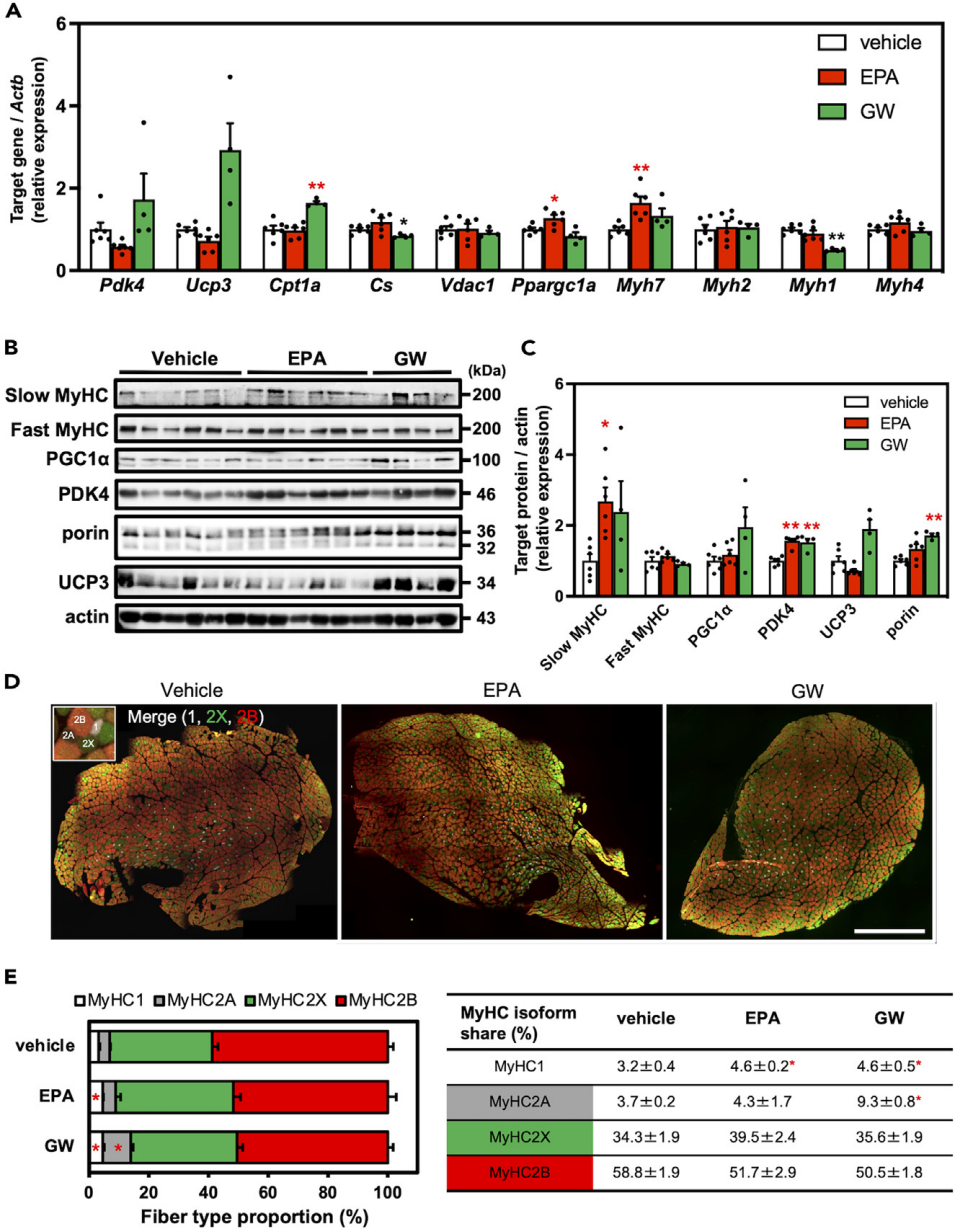
Effects of 4-week EPA supplementation on expression of muscle fiber type related factors in rat skeletal muscles The mRNA (A) and protein (B and C) expression of muscle fiber type related factors in extensor digitorum longus (EDL) muscle of rats administrated with eicosapentaenoic acid (EPA) for 4 weeks. The intensity of the immunoblot bands (B) were quantified and normalized to actin as a loading control (C). Cross-section of plantaris muscle was stained for determination of muscle fiber type (myosin heavy chain (MyHC)1 [white], MyHC2A [unstained], MyHC2X [green], and MyHC2B [red]) (D). The bars indicate 1000 μm. Fiber-type proportions of plantaris muscle of each group (E). Data are means ± SEM (*n* = 6 in vehicle and EPA group, *n* = 4 in GW501516 group, ∗∗*p* < 0.01 and ∗*p* < 0.05 compared with the vehicle controls).

To corroborate the improvement of muscle function following EPA or GW501516 administration, we analyzed the mRNA and protein expression levels of genes related to energy metabolism, mitochondria, and different types of muscle fibers. We used two types of muscle tissues, namely fast-twitch fiber-predominant tissue (EDL and plantaris) and slow-twitch fiber-predominant tissue (soleus) muscles. In terms of both mRNA and protein expression, only EPA administration upregulated MyHC1 (*Myh7*) expression in the EDL muscles, whereas the GW group did not show this increase (Figures 4A–4C). Immunofluorescence microscopy for plantaris muscle cross-sections revealed that EPA administration increased proportion of type 1 (MyHC1-positive) fibers (Figures 4D and 4E), and this result augmented the analysis of mRNA and protein expression levels. Interestingly, the increased type 1 and 2A fibers were observed in GW group compared to the control group.

The mRNA expression *Angptl4 (p < 0.05)*, *Ppargc1a (p < 0.05)*, *Pdk4*, and *Ucp3* decreased in the EDL muscles of the EPA group, whereas the GW group showed the opposite effect (Figure 4A). Regardless of the decreased mRNA expression levels, the protein expression levels of PDK4 increased significantly in the EPA group (Figures 4B and 4C). In soleus muscles, most mRNA and protein expression levels studied did not change with EPA and GW supplementation (Figures S2A–S2C), because changes in expression are sometimes observed with differences in reactivities to drugs or functional foods between tissues that predominantly have fast-twitch or slow-twitch fibers.^7^^,^^26^

### Comprehensive analyses show that EPA regulates both AMPK and PPARδ signaling

To identify the pathways activated after treatment with EPA or GW501516, we comprehensively analyzed mRNA (by RNA sequencing [RNA-seq]) and metabolites (by capillary electrophoresis-triple quadrupole/time-of-flight mass spectrometry [CE-QqQ/TOFMS]) levels in differentiated L6 myotubes.

**Figure 5:**
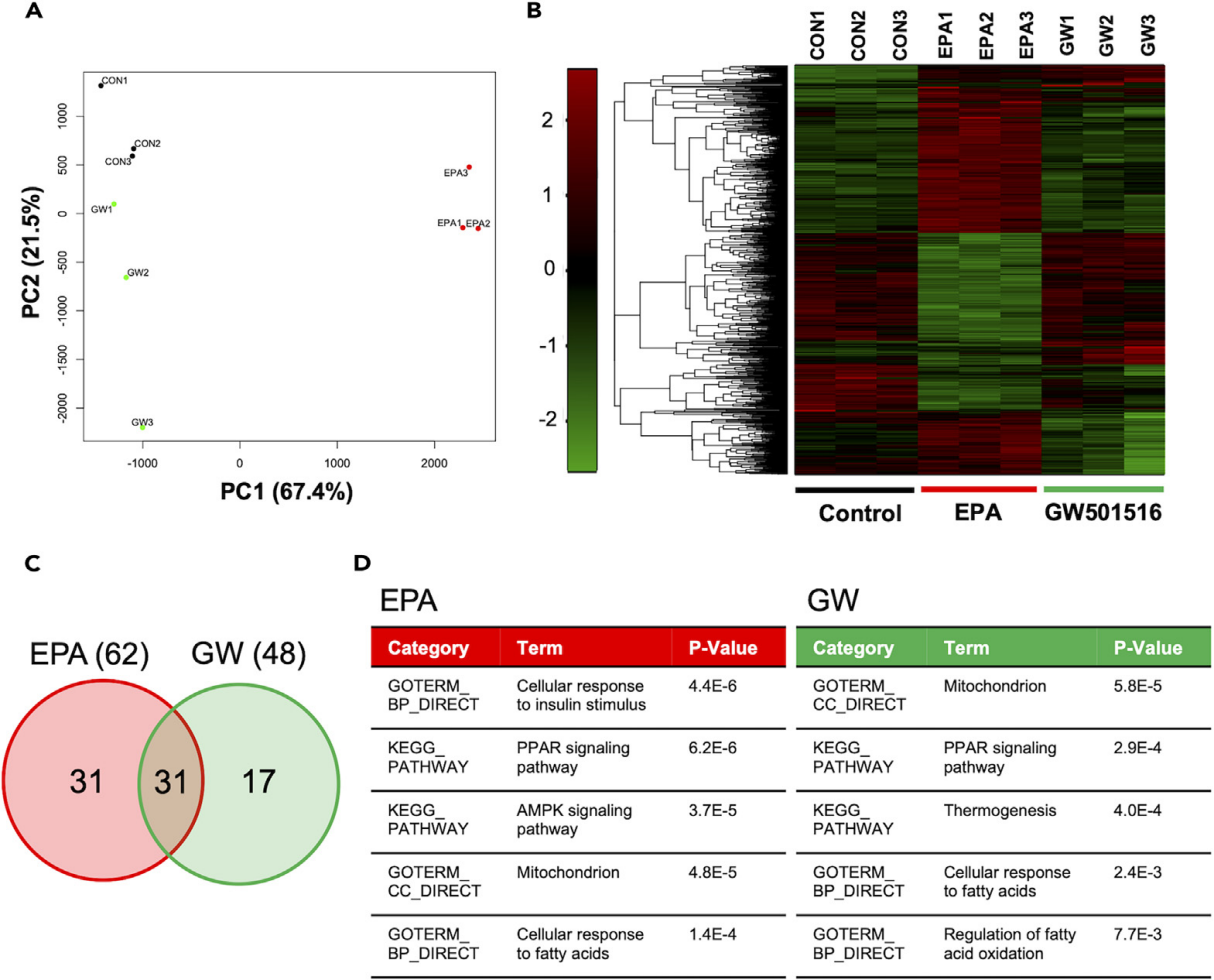
Effects of EPA supplementation on comprehensive mRNA expression in L6 myotubes (A) Score plot of a principal component analysis (PCA) of the data obtained from RNA-seq. Colored dots represent individual samples (*n* = 3 independent cultures, black: control; red: eicosapentaenoic acid (EPA); green: GW501516). (B) Hierarchical clustering analysis (HCA) shown by heatmap of regulated genes in L6 myotubes of control (left, marked by the blue bar), EPA (center, marked by red bar), and GW501516 group (right, marked by green bar). The color-coded scale on the left indicates the normalized mRNA expression. (C) Venn diagram comparing EPA and GW501516 target genes identified in RNA-seq analysis of L6 myotubes (*n* = 3 independent cultures). (D) The differentially expressed genes were classified using gene ontology (GO) functional annotation and the Kyoto Encyclopedia of Genes and Genomes (KEGG) pathway terms.

In the RNA-seq experiment, we identified 32,883 expressed genes. Principal component analysis (PCA) demonstrated clear clustering of the control, EPA, and GW groups (Figure 5A), suggesting that EPA treatment upregulated or downregulated different genes in the muscle cells than GW501516 treatment.

Hierarchical clustering analysis (HCA) revealed a remarkably different pattern among the three groups (Figure 5B). Focusing on the genes regulated by EPA treatment revealed 62 differentially expressed genes (versus the controls), including 42 upregulated (log_2_ fold change >1.0) and 20 downregulated (log_2_ fold change < −1.0) genes. Approximately 50% of the dysregulated genes were common between the EPA and GW501516 groups (Figure 5C), demonstrating that EPA treatment partially mimicked PPARδ agonism in terms of gene expression.

Gene ontology (GO) and pathway analysis of the dysregulated genes revealed significant enrichments in GO terms such as the cellular response to insulin stimulus, PPAR signaling pathway, AMP-activated protein kinase (AMPK) signaling pathway, mitochondria, and cellular response to fatty acids in the EPA group. In mitochondria, the terms PPAR signaling pathway, thermogenesis, cellular response to fatty acids, and regulation of fatty acid oxidation were enriched in the GW group (Figure 5D). EPA treatment strongly promoted the terms cellular response to insulin stimulus and AMPK signaling pathway, based on a comparison with the GW group, suggesting that these pathways were exclusive to EPA treatment. Activated AMPK leads to glucose uptake,^27^ mitochondrial biogenesis, and improved oxidative metabolism,^28^ in accordance with the improved systemic metabolism and muscle function observed in our *in vivo* experiments. The pathways related to mitochondrial and fatty acid metabolism were consistent with those in the GW group.

**Figure 6:**
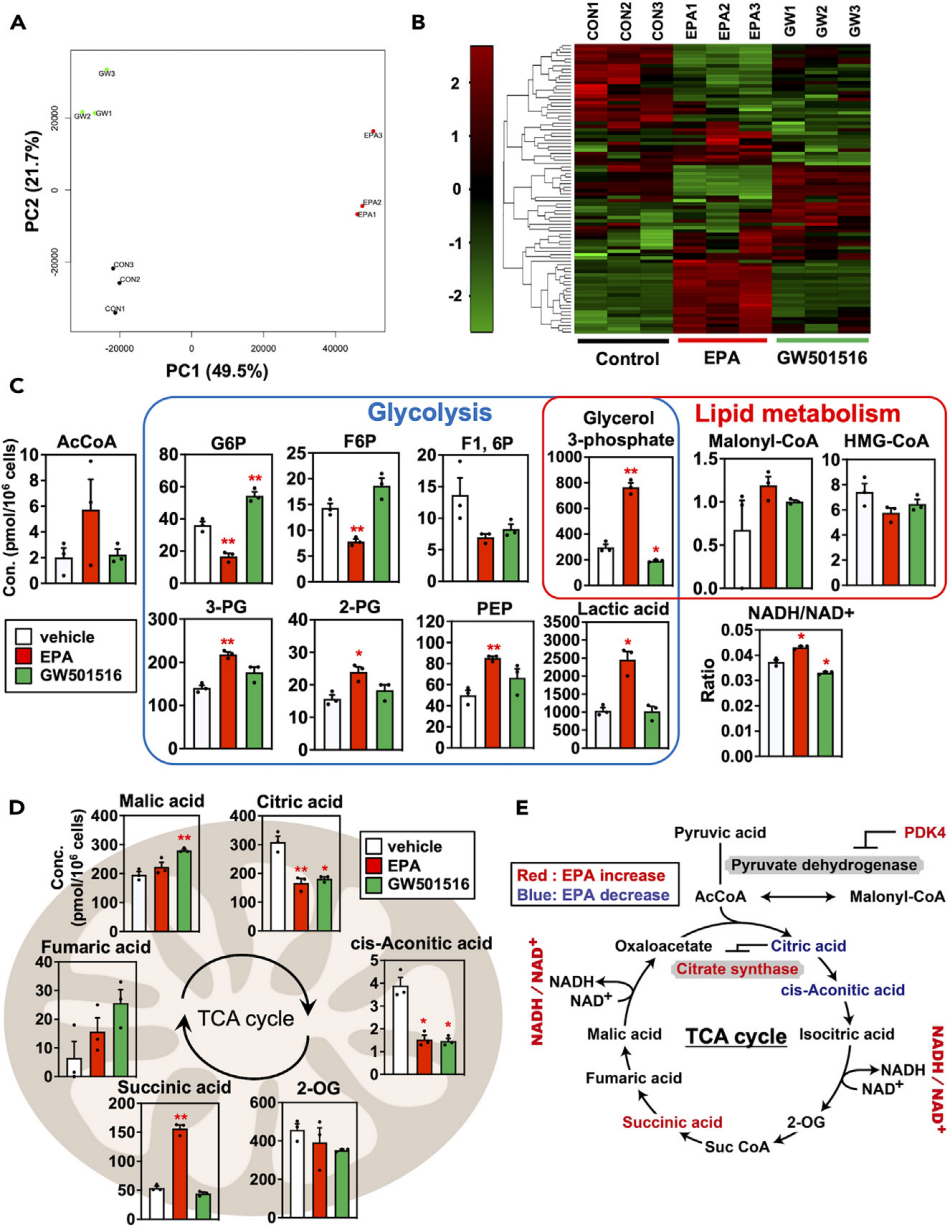
Effects of EPA supplementation on comprehensive metabolites profiling in L6 myotubes (A) Score plot of a principal component analysis (PCA) of the data obtained from metabolome analysis. Colored dots represent individual samples (*n* = 3 independent cultures, black: control; red: eicosapentaenoic acid (EPA); green: GW501516). (B) Hierarchical clustering analysis (HCA) shown by heatmap of regulated metabolites in L6 myotubes of control (left, marked by the blue bar), EPA (center, marked by red bar), and GW501516 group (right, marked by green bar). The color-coded scale on the left indicates the normalized metabolite expression. (C and D) Quantitative analysis of L6 myotube metabolites belonging to energy metabolism (C) and tricarboxylic acid (TCA) cycle (D). (E) Diagram showing the factors related to TCA cycle. Red and blue labels represent up- and downregulation induced by EPA treatment, respectively. Data are means ± SEM (*n* = 3 independent cultures, ∗∗*p* < 0.01 and ∗*p* < 0.05 compared with the vehicle controls).

Our metabolomic analysis revealed 116 detectable peaks (52 cations and 64 anions) when performing CE-QqQ/TOFMS in the anion and cation modes, respectively. The whole metabolite profiles among the control, EPA, and GW groups were clearly distinguishable by PCA and HCA (Figures 6A and 6B). This classification was very similar to that of the transcriptome analysis. To clarify the functional characteristics of different metabolite contents occurring in myotubes after EPA supplementation, we investigated the changes in individual metabolites using the Kyoto Encyclopedia of Genes and Genomes (KEGG) pathway database. The levels of metabolites related to glycolysis and lipid metabolism were shown in Figure 6C. Production of glucose-6-phosphate (G6P) and fructose-6-phosphate (F6P) decreased in the EPA group (versus the control group), whereas production of glycerate 3-phosphate (3-PG), glycerate 2-phosphate (2-PG), phosphoenolpyruvic acid (PEP), and lactic acid increased. The alterations in glycolytic-pathway metabolites differed from those in the GW group. Muscle glycolysis closely regulates AMPK activation,^28^ and we demonstrated that the AMPK pathway was activated in the EPA group by GO analysis, using RNA-seq. AMPK can serve as a cellular-energy sensor and is activated in skeletal muscles during exercise in response to an increase in the (adenosine monophosphate [AMP] + adenosine diphosphate [ADP])/adenosine triphosphate ATP ratio. Our data revealed no significant differences in the adenylate energy charge (AEC) and total adenylate levels among all groups, suggesting that similar energy states were maintained. However, the AMP and ADP levels were elevated in the EPA group, and the (AMP + ADP)/ATP ratio was 1.5-fold higher in the EPA group than in the control group (Figure S3), suggesting AMPK activation. The level of glycerol-3-phosphate, a common metabolite of the glycolytic and lipid oxidation pathways, was increased in the EPA-supplementation group, whereas glycerol-3-phosphate was decreased in the GW group. The ratio of oxidized nicotinamide adenine dinucleotide (NAD^+^) to reduced nicotinamide adenine dinucleotide plus hydrogen (NADH) was higher in the EPA group, which was reversed in the GW group.

Next, we focused on the metabolites related to the tricarboxylic acid (TCA) cycle. The levels of citric acid and *cis*-aconitic acid were lower in the EPA group than in the control group, whereas succinic acid production increased (Figure 6D). The TCA cycle can be promoted when acetyl-CoA (AcCoA) and oxaloacetate are produced by citrate synthase. Citric acid inhibits citrate synthase and the TCA cycle. Citric acid production was significantly lower in myotubes supplemented with EPA or GW501516, suggesting that EPA accelerated the TCA cycle via PPARδ activation. To confirm in detail the phenomenon that occurred with EPA supplementation, the change in factors related to TCA cycle by EPA treatment was summarized in Figure 6E. EPA increased the NADH/NAD^+^ ratio, a main contributor to oxidative phosphorylation-mediated ATP production,^29^ and decreased citric acid, an inhibitor of citrate synthase. The mRNA expression of citrate synthase was upregulated by EPA supplementation in isolated muscle fiber analysis (Figure 2F). PDK4, which was upregulated by EPA (Figures 2A and 2F), inhibits pyruvate dehydrogenase activity, which in turn will increase the influx of AcCoA from beta-oxidation into the TCA cycle, thereby leading to enhanced fatty acid oxidation. Taken together, EPA activated TCA cycle through lipid utilization in the muscle cell, and this change in muscle metabolism may contribute to the increase in oxidative type 1 fibers and systemic oxygen consumption.

## Discussion

**Figure 7:**
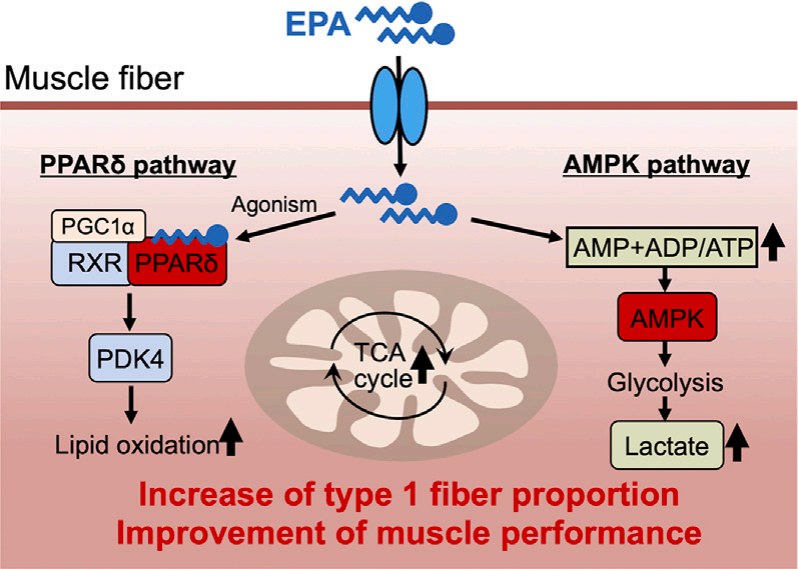
A schematic model of type 1 fiber formation by EPA Eicosapentaenoic acid (EPA) is imported in muscle cells via fatty acid transporters such as FAT/CD36, and then activates AMP-activated protein kinase (AMPK) and peroxisome proliferator-activated receptor (PPAR)δ pathways. Activated AMPK signaling leads to the glycolysis and lactate production. In addition, activated PPARδ promotes lipid oxidation and activates TCA cycle. These alterations of metabolism induce mitochondrial expression and the complicated coordination of AMPK signaling, PPARδ pathways, and metabolism contributes the formation of type 1 fiber.

The main findings of this study are as follows: (1) EPA supplementation for 4 weeks promoted whole-body fat oxidation and improved muscle function accompanied by increased type 1 fiber proportion in rats; (2) EPA functioned as an agonist and activated PPARδ, which upregulated oxidative metabolic genes in muscle cells; (3) comprehensive transcriptomic and metabolomic analyses showed that EPA activated the PPARδ and AMPK pathways, and this combined effect might increase type 1 fiber proportion (Figure 7). Overall, our current findings suggest that EPA is a novel exercise-mimetic food component. The findings demonstrate a fundamental link between nutrition and muscle function and could facilitate the development of novel strategies in health and sports sciences for the improvement of metabolism and promotion of muscle function.

Elucidation of the mechanisms involved in the coordinate regulation of muscle metabolic and structural programs during fiber-type transition has implications for new therapeutic approaches for many human diseases, including metabolic disorders and obesity. Muscle fiber-type composition is influenced by both fundamental developmental mechanisms and postnatal physiological stimuli, such as exercise training.^30^^,^^31^ Previous studies have established that nuclear receptors such as PPARs and ERRs, along with their co-regulators PGC-1s and AMPK, are key regulators of myocytes energy metabolism.^1^^,^^32^^,^^33^ We initially focused on PPARδ as a type 1 fiber inducer because EPA showed high PPARδ ligand activity *in vitro* experiments. PPARδ is closely related to lipid metabolism in skeletal muscle, and muscle-specific VP16-PPARδ transgenic mice drastically increased slow oxidative type 1 fibers.^18^ Gan et al. reported that muscle-specific PPARδ transgenic mice increased ERRγ/β, which activates *miR-208b* and *miR-499* genes and contributes to the regulation of type 1 fibers by suppressing of repressors of type 1 fiber formation such as Sox6.^34^ We did not analyze the downstream of PPARδ activation, and further research is needed to elucidate the detailed mechanisms of EPA-induced muscle fiber type transformation. Interestingly, although data have not been shown, they suggest that activation of the type 1 fiber program by PPARδ occurs by a ligand-independent mechanism based on their *in vitro* study. Consistent with this suggestion, Narkar et al. revealed that treatment with GW501516 alone did not increase type 1 fibers; however, the combination of GW501516 and exercise training resulted in a significant and synergistic increase in type 1 fiber abundance in mice, suggesting that increase of type 1 fiber requires certain factors induced by exercise, in addition to PPARδ activation.^26^ In our *in vivo* study, only EPA supplementation increased MyHC1 expression, the type 1 fiber marker, at both mRNA and protein levels. With GW501516 administration, an increase in MyHC1 was observed only by immunohistochemistry; however, there were no changes in western blotting or qPCR analysis. These findings led us to hypothesize, as in the previous report, that there are other important factors involved in the EPA-induced increase in type 1 fibers in addition to PPARδ activation. Our combined transcriptome and metabolome analyses determined AMPK activation as another key factor simultaneously inducing type 1 fiber formation with PPARδ under EPA supplementation. AMPK is one of the representative kinases activated by aerobic exercise and has profound effects on gene expression and oxidative metabolism in skeletal muscles.^35^^,^^36^ Indeed, mice defective in AMPK signaling in muscles exhibit reduced type 1 fibers and running endurance.^28^ Moreover, PPARδ and AMPK cooperate in the transcriptional regulation of oxidative metabolism-related genes,^26^^,^^37^^,^^38^^,^^39^ suggesting that the two factors play key roles in skeletal muscle metabolism. Collectively, synergistic activation of the PPARδ and AMPK pathways by EPA may induce an increase in type 1 fiber proportion in rat muscles and facilitate adaptive mitochondrial and muscle function.

Several crystal structure studies have revealed the binding modes of ligands in the ligand-binding domain (LBD) of PPAR family (α, γ and δ). Shiraki et al. revealed that 15-deoxy-delta12,14-prostaglandin J2 (15-dPGJ2) forms a covalent bond with the Cys285 residue in the PPARγ LBD.^40^ Then, Waku et al. demonstrated the interaction of endogenous fatty acid derivatives with PPARγ by a covalent binding structure describing cocrystal structure.^41^ Although it has not been shown whether PPARδ also exhibits the same ligand-binding mode as PPARγ, it is possible because GSK3787, an irreversible antagonist of PPARδ, forms a covalent bond with a cysteine residue in the LBD of PPARδ.^42^ Thus, EPA could activate PPARδ through forming a covalent bond with PPARδ. Other molecular interactions are also crucial to the ligand binding of PPARδ. EPA occupies the ligand-binding pocket of PPARδ in two conformations, and a combination of hydrogen bonds and hydrophobic interactions stabilizes the binding of EPA in both conformations.^43^ In particular, the acid group of EPA is oriented toward the activation function of two helices and held in place through a network of hydrogen bonds and hydrophobic interactions with specific amino acid residues. The remarkable conservation of this network of hydrogen bonds suggests that it must be crucial for ligand-mediated activation of PPARδ. In addition, the hydrophobic tail of EPA is stabilized by numerous hydrophobic interactions with PPARδ, which is why PPARδ only binds to fatty acids of a particular chain length, such as EPA (C20). Short-chain fatty acids (C < 14) could not make the hydrophobic interactions required for stable ligand binding. Long-chain fatty acids (C > 20) could not fit into the pocket in either docking mode and would be exposed to the destabilizing effects of the solvent. This report supports our finding that EPA, not DHA, is the primary active component in fish oil, stimulating muscle fiber type conversion via PPARδ.

Mitochondria are the key sites of aerobic energy metabolism and, in skeletal muscle, are the greatest contributor to the superior endurance of slow-twitch muscle fibers. Activation of AMPK and PGC-1α, initiated by endurance exercise training, promotes mitochondrial biosynthesis,^44^ and the function of lactic acid, a product of the anaerobic glycolytic system, has recently attracted attention. It had been argued that muscle fatigue and lactic acid accumulation are basically synonymous; however, the role of lactic acid in muscle fatigue is being explored in depth, and Allen et al. reported that lactic acid is “the latest performance-enhancing drug.”^45^ Intracellular lactate levels are determined by the balance between the rate of glycolysis and mitochondrial oxidation;^46^ however, because the maximum glycolysis rate is higher than the rate of oxidation, lactic acid is produced by the breakdown of glucose.^47^ Although not fully understood, it has also been suggested that mitochondrial mass increases to adapt to elevated lactic acid levels.^48^^,^^49^^,^^50^ Tanida et al. reported that lactic acid ensures mitochondrial biogenesis via the LRPGC1/ERRγ-TFAM pathway in liver cells.^51^ Based on these reports and our comprehensive analysis, we hypothesized that EPA administration induces high lactic acid levels in muscle cells, resulting in activated glycolysis, enhanced mitochondrial pathways, and improved muscle function. We believe that these specific metabolic changes were associated with the fiber-type alterations and complicated coordination of the AMPK and PPARδ pathways, as described previously.

In conclusion, our study revealed that EPA, a unique fatty acid in fish oil, improves muscle function and oxidative metabolism, with an increase in type 1 muscle fiber proportion in rats. Our comprehensive transcriptomic and metabolomic analyses suggest that EPA affects both the PPARδ and AMPK pathways. Collectively, our current findings suggest that EPA is a novel exercise-mimetic food component that could improve metabolism and promote muscle function. The global obesity epidemic and its associated chronic diseases are largely attributed to an imbalance between caloric intake and energy expenditure. Although physical exercise remains the best solution for obesity, the development of muscle-targeted “exercise mimetics” may soon provide a pharmaceutical alternative for the management of an increasingly sedentary lifestyle.

### Limitations of the study

Our study was based on the changes in muscle fiber type compositions induced by dietary fish oil intake and the fact that fish oil induced type 2B-to-2X changes in a previous study.^7^ In the present study, we identified EPA as a functional component of fish oil and that it increases the abundance of type 1 fibers; however, we could not clarify why fish oil and EPA regulate different muscle-fiber types. Gong et al. reported that dietary fish oil intake increased the proportion of type 1 muscle fibers in pigs.^52^ They used a diet containing 3.5% fish oil for pigs, whereas in our previous study, we used a diet containing 15% fish oil for rats. In terms of mechanism, Gong et al. reported that fish oil activated calmodulin-dependent protein kinase (CaMK) II pathway which closely related to AMPK activation. On the other hand, because the transcriptional coactivator PGC-1β drives the formation of type 2X fibers,^53^ the phenomenon observed by fish oil intake in our previous study may be different from the EPA-induced activation of PPARδ and AMPK pathways that we have demonstrated here. This suggests that the effect of fish oil intake on muscle fiber types varies and is influenced by the differences in the concentration of bioactive components and animal species. The discrepancy could also be attributed to the difference between complex food components (fish oil) and single ingredients (EPA). Multiple ingredients, not just a single ingredient, are ingested when consuming foods, and fish oil is composed of multiple fatty acids that are metabolized into several important components, such as eicosanoids. Although the complexity of food ingredients may mask the effects of such single ingredients, combinations of several food ingredients could enhance or suppress their physiological effects. For example, vitamin A enhanced the antitumor effect of green tea polyphenols in individuals with melanoma^54^ and saturated fatty acids attenuated the anti-obesity effect of green tea extracts in white adipose tissue.^55^ Dietary leucine and fish oil supplementation cooperatively regulated muscle fiber transformation and increased the proportion of type 1 fibers and mitochondrial biogenesis in piglet muscle.^52^ Therefore, we selected DHA as an enhancer of the EPA effect because DHA shows high affinity as a ligand for the retinoid X receptor, which forms heterodimers with PPARδ.^56^^,^^57^ However, combined treatment with EPA and DHA did not exhibit synergistic effects on Pdk4 expression in muscle fiber (Figure S4). Recent data have shown that the metabolites of EPA and DHA elicit various biological activities, including functioning as PPARδ activators.^58^ Thus, we are interested in focusing on the functionality of EPA with food pairing in addition to the effects of single food components in the future. Another limitation of this study is the discrepancy between the results obtained from *in vitro* and *in vivo* experiments. This suggests that factors external to muscle cells, such as motor nerves and physical stimulation, may contribute to the increase in type 1 fibers induced by EPA. Additionally, circulating hormones and other factors secreted from non-muscle tissues could also contribute to these differences. A detailed study of the EPA-induced crosstalk between muscle and other organs would resolve this inconsistency.

## STAR★Methods

### Key resources table

**Table:** 

REAGENT or RESOURCE	SOURCE	IDENTIFIER
**Antibodies**		

Mouse monoclonal anti-actin	Chemicon	Cat#MAB1501 (clone C4) RRID: AB_2223041
Mouse monoclonal anti-porin	Abcam	Cat#Ab14734 (clone 20B12AF2) RRID: AB_443084
Mouse monoclonal anti-slow MyHC	Sigma-Aldrich	Cat#M8421 (clone NOQ7.5.4D) RRID: AB_477248
Mouse monoclonal anti-fast MyHC	Sigma-Aldrich	Cat#M4276 (clone MY-32) RRID: AB_477190
Rabbit polyclonal anti-PDK4	Proteintech	Cat#12949-1-AP RRID: AB_2161499
Rabbit polyclonal anti-UCP3	Abcam	Cat#Ab3477 RRID: AB_2304253
Rabbit polyclonal anti-PGC1α	Calbiochem	Cat#516557 RRID: AB_2268432
Mouse monoclonal anti-MyHC1	Sawano et al., 2016^59^	clone 4B51E8
Mouse monoclonal anti-MyHC2X	Sawano et al., 2016^59^	clone 6F12H3
Mouse monoclonal anti-MyHC2B	Sawano et al., 2016^59^	clone 2G72F10

**Biological samples**		

Rat skeletal muscle	This paper	N/A

**Chemicals, peptides, and recombinant proteins**		

Palmitic acid	Sigma-Aldrich	Cat#P0500
Oleic acid	Nacalai tesque	Cat#25630-51
Linoleic acid	Sigma-Aldrich	Cat#L1012
Arachidonic acid	Sigma-Aldrich	Cat#A3611
Linolenic acid	Sigma-Aldrich	Cat#L2376
Eicosapentaenoic acid	Cayman	Cat#90110
Docosahexaenoic acid	Cayman	Cat#90310
GW501516	LC Laboratories	G-4789
GSK0660	Cayman	Cat#15272
WY14643	Sigma-Aldrich	Cat#C7081
Rosiglitazone	Sigma-Aldrich	Cat#R2408

**Oligonucleotides**		

See Table S2 for primer sequences.		

**Critical commercial assays**		

EnBio RCAS for PPARδ	Fujikura Kasei	Cat#PPARD-CBP

**Deposited data**		

Raw and analyzed data (RNAseq)	This paper	DRA: DRA017310
Raw and analyzed data (metabolome)	This paper	Table S3 and Metabolomics Workbench: Available upon request

**Experimental models: Cell lines**		

Rat: L6 cells	Provided by laboratory of muscle and meat science in Kyushu University	N/A
Rat: FDB-derived Isolated muscle fiber	Komiya et al., 2015^25^	N/A

**Software and algorithms**		

ImageJ	NIH	Version 1.52
GraphPad Prism	GraphPad	Version 10.0.0
R	R Core Team	Version 4.1.3
SPSS	IBM	Version 29.0.1
HISAT2	Kim et al., 2015^60^	HISAT 2.2.1
featureCounts	Liao et al., 2014^61^	subread-2.0.6
edgeR	McCarthy et al., 2012^62^	3.36.0
heatmap.2	https://cran.r-project.org/web/packages/gplots/index.html	gplots 3.1.2
DAVID Bioinformatics	https://david.ncifcrf.gov/tools.jsp	Version 6.8

**Other**		

ARCO2000	Arcosystem	ARCO-2000-RAT
KFWS Series Small-sized Waterproof Foil Strain Gages	KYOWA	KFWS-2N-120-C1-11L5M2R
DB-A Bridge Box	KYOWA	DB-120A
Instrumentation Amplifier	KYOWA	WGA-101AS1

### Resource availability

#### Lead contact

Further information and requests for resources and reagents should be directed to and will be fulfilled by the lead contact, Yusuke KOMIYA (komiya@vmas.kitasato-u.ac.jp).

#### Materials availability

This study did not generate unique new reagents or mouse lines.

#### Data and code availability

• The complete sequencing data of the L6 myotube reported in this paper is deposited at the DNA DataBank of Japan Sequence Read Archive (DRA) (www.ddbj.nig.ac.jp/) and the accession number is listed in the key resources table. The metabolites dataset generated and analyzed during the current study is publicly available at the Metabolomics Workbench (https://www.metabolomicsworkbench.org) and the raw data is shown in Table S3. The accession number of metabolome analysis is available from the lead contact upon request. The datasets that support the findings of this study are available in this article and the supplemental data.
• This work does not report original code.
• Any additional information required to reanalyze the data reported in this paper is available from the lead contact upon request.

### Experimental model and study participant details

#### Animal experiments

All experiments involving animals were conducted in strict accordance with the recommendations in the Guidelines for Proper Conduct of Animal Experiments published by the Science Council of Japan and ethics approvals from the Kyushu University Institutional Review Board (approval No. A22–148, A24–138, A26–081, and A28–092). Male Fischer F344 rats (purchased from KBT Oriental, Tosu, Japan at 6-wks old) were housed at 22 ± 2°C and 55 ± 10% humidity on a 12:12 light/dark cycle and received water and regular food (CRF-1, Oriental Yeast, Tokyo, Japan) *ad libitum*. Rats were divided into three groups: vehicle (0.5% carboxymethyl cellulose diluted in saline); EPA group (1000 mg/kg/day EPA ethyl ester) or GW group (10 mg/kg/day GW501516) and each reagent was orally administrated for 4 weeks. The dose of EPA and GW501516 was determined by preliminary experiment (Figure S5A) and published data,^18^ respectively. After 4-week administration period, rats were subjected to respiratory gas analysis and measurement of maximum muscle-contraction force and endurance. The soleus, extensor digitorum longus (EDL), and plantaris muscles were harvested one day after the measurement of muscle performance. The growth performance and tissue weights were shown in Table S1.

#### Cell lines

The L6 cells suspension was seeded into 12-well plates coated with a collagen at a cell density of 5.0 × 10^3^ cells per well and maintained for 24 hours in growth medium [DMEM supplemented with 10% FBS, 1% antibiotic-antimycotic mixed stock solution, and 0.5% gentamicin]. After reaching confluence, the cells cultured in differentiation medium [DMEM supplemented with 2% horse serum (HS; Invitrogen), 1% antibiotic-antimycotic mixed stock solution, and 0.5% gentamicin] for 10 days to form myotubes.

#### Primary cell cultures

The muscle fibers were isolated and cultured according to previous report.^25^ Briefly, flexor digitorum brevis (FDB) muscles were harvested from male F344 rats (3-4 week-old), and were incubated for 90 min in collagenase solution containing 0.2% collagenase type 1 (Worthington, USA), 0.1% elastase (Worthington), 0.0625% protease from Streptomyces griseus (Sigma–Aldrich, USA), 0.033% dispase (Invitrogen, USA), and 10% fetal bovine serum (FBS, Invitrogen) in physiological rodent saline. After collagenase digestion, muscle tissues were triturated with fire-polished pipette to separate bundles to single fibers. Isolated fibers were cultured in maintainance medium [DMEM supplemented with 20% serum replacement (Invitrogen), 1% antibiotic-antimycotic mixed stock solution, and 0.5% gentamicin].

### Method details

#### Respiratory gas analysis

The rats were individually transferred to acrylic chambers (120 × 150 × 240 mm) for 2 h before start the measurement to acclimate them to the experimental environment. The last administration of EPA or GW501516 was finished at least twelve-hour before measurement to avoid acute effects. The respiratory quotient (RQ), carbohydrate oxidation and fat oxidation were computed on the basis of VO_2_ and CO_2_ production. Especially, carbohydrate and fat oxidation were calculated using the stoichiometric equations of Frayn.^63^ Gas analysis was performed using an open circuit metabolic gas analysis system connected directly to a mass spectrometer (ARCO-2000; Arco System Inc., Chiba, Japan) for 4h in the light period. Room air was pumped through the chambers at a rate of 0.3 L/min. Expired air was dried in a cotton-thin column and then directed to an O_2_/CO_2_ analyzer for mass spectrometry. Rats were received water *ad libitum* but were prohibited from access to food during respiratory gas analysis.

#### Maximum muscle-contraction force and endurance

The measurement of maximum muscle-contraction force and endurance was conducted by nerve-stimulation protocol according to Iwata et al.^64^ with some modifications.^65^ Maximum isometric planter-flexion force was measured from right hind-limb muscles under anesthesia with continuous 4% sevoflurane exposure (fluoromethyl 2,2,2, -trifluoro-1-(trifluoromethyl) ethyl ether; Maruishi Pharmaceutical, Osaka, Japan). Briefly, a set of four strain gauges (KFWS-2N-120-C1–11, Kyowa Electronic Instruments, Tokyo, Japan) attached to the footplate was connected to an instrumentation amplifier (WGA-101AS1; Kyowa Electronic Instruments) and a MacLab/4S digital converter (ADInstruments, Colorado Springs, CO). Tetanic contraction force of the posterior muscle in the lower hind limb (right side) was generated by electrical stimulations with an electronic stimulator module (SEN-5201 and SEN-3201; Nihon Kohden, Tokyo, Japan) via a bipolar hook-shaped electrode (KS207-024; Unique Medical, Tokyo, Japan) on tibial nerve. Muscle force was evaluated by successive electrical stimulations every 1 sec, and force was recorded for 100 sec (100 times stimulus) and converted to torque by multiplying the length between the medial malleolus and the head of the first metatarsal bone for each rat. The stimulation conditions (amplitude 60V, rectangular pulse width 1 ms, duration 160 ms with intervals of 3 ms after each pulse, frequency 250 Hz) were optimized to generate maximum isometric force at the initial.

#### Reagents

We used 30 μM fatty acids in all *in vitro* study. Fatty acids were not conjugated to albumin. In preliminary experiments, we confirmed a similar reaction between free fatty acids and albumin-conjugated fatty acids. The concentrations of fatty acids and EPA were determined according to the preliminary experiment (Figure S5B) and a ref.^17^ Fatty acids used in this study were listed: palmitic acid, oleic acid, linoleic acid, arachidonic acid, linolenic acid, eicosapentaenoic acid, and docosahexaenoic acid. The reagents for PPARs were listed: 100 nM GW501516 (a PPARδ selective agonist), 800 nM GSK0660 (a PPARδ antagonist), 100 nM WY14643 (a PPARα selective agonist), and 100 nM rosiglitazone (a PPARγ selective antagonist). Isolated fibers or differentiated myotubes were treated with fatty acids or these reagents for 12h in qPCR and RNA-seq analysis, for 96h in metabolome analysis, and for 120h in Western blotting analysis and immunocytochemistry.

#### PPARδ ligand assay

The PPARδ ligand activity of fatty acids was measured by using a nuclear receptor cofactor assay system (EnBio RCAS for PPARδ; Fujikura Kasei Co. Ltd., Tokyo, Japan) according to the manufacturer’s instructions. The intensity of the GW501516 treatment was set at 100% (B/B_max_), and relative intensity is presented as the fold induction relative to that of the GW501516.

#### Luciferase reporter assay

The luciferase ligand assay was performed using a highly sensitive system, which was developed by modifying the Dual-luciferase® Reporter Assay System (Promega, WI, USA), as described previously.^66^ Briefly, for the assay using the GAL4/PPAR chimera system, we transfected p4xUASg-tk-luc (a reporter plasmid); pM-hPPARδ (expression plasmids for chimera proteins containing a GAL4 DNA binding domain and one of the human PPAR ligand binding domains); and pRL-CMV (an internal control) into CV1 cells cultured in a 96-well plate. The transfection was performed using LipofectamineTM2000 (Invitrogen, Corp., Carlsbad, CA, USA) according to the manufacturer’s protocol. Five hours after the transfection, the transfected cells were cultured in medium containing each fatty acid for an additional 16 h. The luciferase ligand assay was performed using the dual luciferase system in accordance with the manufacturer’s protocol.

#### RNA isolation and RT-qPCR

Total RNA was extracted from EDL muscle, soleus muscle, isolated fibers, and L6 cells with Trizol reagent (Thermo Fisher Scientific Inc.), and reverse transcribed using SuperScript III Reverse Transcriptase (Invitrogen) and Oligo d(T)16 primer (Applied Biosystems, Waltham, MA, USA) according to the manufacturer’s instructions. RT-qPCR using the LightCycler 1.5 (Roche Diagnostics, Switzerland) was performed by the intercalator method using specific primers and EvaGreen dye (Biotium, USA). Amplicon specificity was verified by melting curve analysis. All primers were designed using the ProbeFinder software (version 2.53; Roche Diagnostics) with an intron-spanning assay. The primer sets used in this study are listed in Table S2. Hypoxanthine–guanine phosphoribosyl transferase (HPRT) was used as a reference gene.

#### Western blotting

Crashed muscles (EDL and soleus muscle, approximately 50 mg) or L6 cells were homogenized in SDS solution containing 10% SDS, 40 mM DTT, 5 mM EDTA, and 0.1 M Tris-HCl buffer (pH 8.0), in which a protease inhibitor cocktail (Protease Inhibitor Cocktail for Use with Mammalian Cell and Tissue Extracts, Nacalai Tesque, Inc., Kyoto, Japan) was added at 1:100. The sample homogenates were heated in boiling water for 3 min. Total protein concentrations were determined using Pierce BCA Protein Assay Reagent (Thermo Fisher Scientific, Waltham, MA, USA) and finalized at 8 μg/μL. The prepared protein samples were separated via 10% SDS gel electrophoresis under reducing conditions and transferred onto PVDF membranes (Bio-Rad, Hercules, CA, USA). The membranes were then incubated with a blocking reagent (5% powdered skim milk in TTBS) for 45 min before incubation with primary antibodies diluted in CanGetSignal solution 1 (Toyobo, Osaka, Japan) overnight at 4°C. The following antibodies were used: mouse monoclonal anti-actin (Chemicon MAB1501 (clone C4), 1:10000); anti-porin (Abcam ab14734, 1: 2000); anti-slow MyHC (Sigma-Aldrich M8421 (clone NOQ7.5.4D), 1:1000); anti-fast MyHC (Sigma-Aldrich M4276 (clone MY-32), 1:1000); rabbit polyclonal anti-PDK4 (Proteintech, 12949-1-AP, 1:2000), and anti-UCP3 (Abcam, ab3477, 1:2000) anti-PGC1α (Calbiochem 516557, 1:2000). The membranes were then incubated for 1 h with a peroxidase-conjugated anti-mouse IgG (Jackson ImmunoResearch 287695, 1:5000) or anti-rabbit IgG secondary antibody (Dako, Santa Clara, CA, USA, 1:5000) diluted in CanGetSignal solution 2 (Toyobo). The bands were detected using enhanced chemiluminescence (ECL; GE Healthcare, Chicago, IL, USA), and images were captured using a Fusion SL-4 chemiluminescence imager (Vilber Lourmat, Marne La Vallée, France). The intensity of the bands was quantified using ImageJ software and normalized to actin as a loading control.

#### Immunocytochemistry

Differentiated L6 myotubes were fixed with 4% paraformaldehyde in PBS for 15 min at 4°C, treated with 0.2% Triton X-100 for 15 min, and then blocked with 3% bovine serum albumin (BSA) in T-PBS for 1 hour at room temperature. Each well was then incubated with primary antibodies against slow MyHC (Sigma-Aldrich M8421 (clone NOQ7.5.4D), 1:100) and fast MyHC (Sigma-Aldrich M4276 (clone MY-32), 1:100) overnight at 4°C. The cells were incubated with a rhodamine-labeled affinity-purified anti-mouse IgG secondary antibody (KPL as SeraCare; Gaithersburg, MD, USA, 1:250) for 1 h at room temperature and then mounted with ProLong™ Diamond Antifade Mountant with DAPI (Thermo Fisher Scientific). The cells were observed under a microscope (BZ-X 810; KEYENCE, Osaka, Japan). To count the each MyHC positive fiber, five images were randomly captured per well. The number of counted fibers was standardized to the area.

#### Immunohistochemistry

Four MyHC isoforms were determined by “stained glass-like staining” reported by Sawano et al.^59^ Briefly, plantaris muscle sections were fixed with steam for 5 minutes, and the slides were then incubated in 1.0% Triton X- 100 in PBS at room temperature for 10 minutes. Subsequently, the slides were incubated with blocking solution (containing 2% donkey serum, 1% bovine serum albumin (BSA), 0.1% cold fish skin gelatin, 0.1% Triton X-100, 0.05% Tween 20, 0.01% avidin, 100 mM glycine, and 0.05% sodium azide in PBS, pH 7.2) for 1 hour at room temperture before incubation overnight at 4°C in the mixture of primary mAbs against MyHC1 (clone 4B51E8) (slow) and MyHC2X (clone 6F12H3), MyHC2B (clone 2G72F10) (fast) (prelabeled with Molecular Probes Alexa Fluor 647, Fluorescein 500, and HyLyte Fluor 594, respectively; 1:100 dilution in the sterile solution containing 1% BSA, 0.1% cold fish skin gelatin, 0.5% Triton X-100, 0.05% Tween 20, 0.01% biotin, and 0.05% sodium azide in PBS). Sections were mounted in VECTASHIELD Antifade Mounting Medium (Vector Lab., Burlingame, CA) and observed under a Leica DMI6000B- AFC fluorescence microscope equipped with a DFC365FX digital camera and LAS AF 3.1.0 software that controls a Tile-Scan program. Slow fibers (type 1) and fast fibers (types 2A, 2X, and 2B) were then counted.

#### RNA-seq library construction and sequencing

Total RNA was extracted from L6 myotubes (3 replicates per group) using the RNeasy Micro Kit (74004, Qiagen, Hilden, Germany) and QIAshredder (79654, Qiagen) according to the manufacturer’s protocol. The extracted total RNA was submitted to Macrogen Japan NGS Service (Tokyo, Japan). Then, RNA-seq libraries were prepared by Truseq stranded mRNA Library (Illumina) and obtained cDNA were subjected to 100-bp paired-end sequencing on Illumina NovaSeq6000 platform to a depth of 40.23–57.98 million reads per sample. The reads were aligned to rat reference genome (Ensembl Rnor6.0) using HISAT2.^60^ The mapped reads were counted with featureCounts,^61^ and differentially expressed genes were analyzed using edgeR with multiple comparison method.^62^ Principal component analysis (PCA) was performed by the prcomp function in R and hierarchical cluster analysis (HCA) was performed by the heatmap.2 package in R. A false discovery rate (FDR) < 0.01 was used to define differentially expressed transcripts between two groups. Genes with differential expression were then subjected to the gene ontology (GO) and pathway analysis performed by Database for Annotation, Visualization and Integrated Discovery (DAVID) to infer the functional roles and relationships.^67^

#### Metabolome analysis

The cells were treated with methanol and incubated at room temperature for 30 sec to suppress enzyme activity after twice rinse with 5% mannitol solution. Next, internal standards (H3304-1002, Human Metabolome Technologies, Inc. (HMT), Tsuruoka, Yamagata, Japan) was added to the cell extract, followed by further incubation at room temperature for 30 sec. The cell extract was then centrifuged at 2,300 ×*g*, 4°C for 5 min, after which the supernatant was centrifugally filtered through a Millipore 5-kDa cutoff filter (UltrafreeMC-PLHCC, HMT) at 9,100 ×*g*, 4°C for 120 min to remove macromolecules. Subsequently, the filtrate was evaporated to dryness under vacuum and reconstituted in 50 μL of mQ water for metabolome analysis at HMT. Analysis was conducted according to HMT’s *C-SCOPE* package, using capillary electrophoresis time-of-flight mass spectrometry (CE-TOFMS) for cation analysis and CE-tandem mass spectrometry (CE-MS/MS) for anion analysis. The time-of-flight mass spectrometer was scanned from m/z 50 to 1,000 and the triple quadrupole mass spectrometer was used to detect compounds in dynamic MRM mode. Peaks were extracted using MasterHands, automatic integration software (Keio University, Tsuruoka, Yamagata, Japan)^68^ and MassHunter Quantitative Analysis B.04.00 (Agilent Technologies) to obtain peak information including *m/z*, peak area, and migration time (MT). Signal peaks were annotated according to HMT’s metabolite database based on their *m*/*z* values and MTs. The peak area of each metabolite was normalized to internal standards, and metabolite concentration was evaluated by standard curves with three-point calibrations using each standard compound. Overall, 116 compounds were measured. Hierarchical cluster analysis (HCA) and principal component analysis (PCA)^69^ were performed by HMT’s proprietary MATLAB and R programs, respectively. Detected metabolites were plotted on metabolic pathway maps using VANTED software.^70^

### Quantification and statistical analysis

Data were expressed as means ± standard errors (SE). Data were firstly analyzed with Levene’s test for equality of variance and p < 0.05 was considered as unequal variance. In the case of equal variances, the Dunnett’s test was used to test differences between group means and control values. In the case of unequal variances, Dunnett-T3 test was used instead of Dunnett’s test. A p < 0.05 was considered statistically significant throughout the study, and statistically significant differences with p < 0.05 and p < 0.01 are indicated in figures by ∗ and ∗∗, respectively. In the experiment of co-supplementation of EPA and PPARδ antagonist (Figure 1D), a Tukey–Kramer test was used to perform multiple comparison. IBM SPSS Statistics (IBM Co., Armonk, NY, U.S.A.) was used for a Dunnett-T3 test. The Excel-Toukei (Social Survey Research Information Co., Ltd., Tokyo, Japan) was used for Levene’s test, Dunnett’s and Tukey–Kramer tests. The graphs were created using Microsoft Excel (Redmond, WA, USA) and GraphPad Prism software (GraphPad Software Inc., San Diego, CA, USA).

## Supplemental information

Document S1. Figures S1–S5 and Tables S1 and S2

Table S3. Absolute quantificative values of metabolites, related to Figures 6 and S3

## References

[1] Baskin K.K., Winders B.R., Olson E.N. (2015). Muscle as a ‘mediator’ of systemic metabolism. Cell Metabol..

[2] Peçanha T., Goessler K.F., Roschel H., Gualano B. (2020). Social isolation during the COVID-19 pandemic can increase physical inactivity and the global burden of cardiovascular disease. Am. J. Physiol. Heart Circ. Physiol..

[3] Lawhun Costello V., Chevance G., Wing D., Mansour-Assi S.J., Sharp S., Golaszewski N.M., Young E.A., Higgins M., Ibarra A., Larsen B., Godino J.G. (2021). Impact of the COVID-19 pandemic on pbjectively measured physical activity and sedentary behavior among overweight young adults: yearlong longitudinal analysis. JMIR Public Health Surveill..

[4] Schiaffino S., Reggiani C. (2011). Fiber types in mammalian skeletal muscles. Physiol. Rev..

[5] Zierath J.R., Hawley J.A. (2004). Skeletal muscle fiber type: Influence on contractile and metabolic properties. PLoS Biol..

[6] Hawley J.A., Hargreaves M., Joyner M.J., Zierath J.R. (2014). Integrative biology of exercise. Cell.

[7] Mizunoya W., Iwamoto Y., Shirouchi B., Sato M., Komiya Y., Razin F.R., Tatsumi R., Sato Y., Nakamura M., Ikeuchi Y. (2013). Dietary fat influences the expression of contractile and metabolic genes in rat skeletal muscle. PLoS One.

[8] Botolin D., Wang Y., Christian B., Jump D.B. (2006). Docosahexaneoic acid (22:6,n-3) regulates rat hepatocyte SREBP-1 nuclear abundance by Erk- and 26S proteasome-dependent pathways. J. Lipid Res..

[9] Scorletti E., Byrne C.D. (2018). Omega-3 fatty acids and non-alcoholic fatty liver disease: Evidence of efficacy and mechanism of action. Mol. Aspect. Med..

[10] Kim M., Goto T., Yu R., Uchida K., Tominaga M., Kano Y., Takahashi N., Kawada T. (2015). Fish oil intake induces UCP1 upregulation in brown and white adipose tissue via the sympathetic nervous system. Sci. Rep..

[11] Mason R.P., Libby P., Bhatt D.L. (2020). Emerging mechanisms of cardiovascular protection for the omega-3 fatty acid eicosapentaenoic acid. Arterioscler. Thromb. Vasc. Biol..

[12] Satoh N., Shimatsu A., Kotani K., Sakane N., Yamada K., Suganami T., Kuzuya H., Ogawa Y. (2007). Purified eicosapentaenoic acid reduces small dense LDL, remnant lipoprotein particles, and c-reactive protein in metabolic syndrome. Diabetes Care.

[13] Tani S., Nagao K., Matsumoto M., Hirayama A. (2013). Highly purified eicosapentaenoic acid may increase low-density lipoprotein particle size by improving triglyceride metabolism in patients with hypertriglyceridemia. Circ. J..

[14] Kamolrat T., Gray S.R. (2013). The effect of eicosapentaenoic and docosahexaenoic acid on protein synthesis and breakdown in murine C2C12 myotubes. Biochem. Biophys. Res. Commun..

[15] Woodworth-Hobbs M.E., Hudson M.B., Rahnert J.A., Zheng B., Franch H.A., Price S.R. (2014). Docosahexaenoic acid prevents palmitate-induced activation of proteolytic systems in C2C12 myotubes. J. Nutr. Biochem..

[16] Le Guen M., Chaté V., Hininger-Favier I., Laillet B., Morio B., Pieroni G., Schlattner U., Pison C., Dubouchaud H. (2016). A 9-wk docosahexaenoic acid-enriched supplementation improves endurance exercise capacity and skeletal muscle mitochondrial function in adult rats. Am. J. Physiol. Endocrinol. Metab..

[17] Forman B.M., Chen J., Evans R.M. (1997). Hypolipidemic drugs, polyunsaturated fatty acids, and eicosanoids are ligands for peroxisome proliferator-activated receptors alpha and delta. Proc. Natl. Acad. Sci. USA.

[18] Wang Y.X., Zhang C.L., Yu R.T., Cho H.K., Nelson M.C., Bayuga-Ocampo C.R., Ham J., Kang H., Evans R.M. (2004). Regulation of muscle fiber type and running endurance by PPARδ. PLoS Biol..

[19] Chen W., Gao R., Xie X., Zheng Z., Li H., Li S., Dong F., Wang L. (2015). A metabolomic study of the PPARδ agonist GW501516 for enhancing running endurance in Kunming mice. Sci. Rep..

[20] Rivera C.N., Hinkle J.S., Watne R.M., Macgowan T.C., Wommack A.J., Vaughan R.A. (2023). PPAR β/δ agonism with GW501516 increases myotube PGC-1 α content and reduces BCAA media content independent of changes in BCAA catabolic enzyme expression. PPAR Res..

[21] Goto T., Takahashi N., Kato S., Egawa K., Ebisu S., Moriyama T., Fushiki T., Kawada T. (2005). Phytol directly activates peroxisome proliferator-activated receptor α (PPARα) and regulates gene expression involved in lipid metabolism in PPARα-expressing HepG2 hepatocytes. Biochem. Biophys. Res. Commun..

[22] Degenhardt T., Saramäki A., Malinen M., Rieck M., Väisänen S., Huotari A., Herzig K.H., Müller R., Carlberg C. (2007). Three members of the human pyruvate dehydrogenase kinase gene family are direct targets of the peroxisome proliferator-activated receptor β/δ. J. Mol. Biol..

[23] Krey G., Braissant O., L'Horset F., Kalkhoven E., Perroud M., Parker M.G., Wahli W. (1997). Fatty Acids, eicosanoids, and hypolipidemic agents identified as ligands of peroxisome proliferator-activated receptors by coactivator-dependent receptor ligand assay. Mol. Endocrinol..

[24] Komiya Y., Anderson J.E., Akahoshi M., Nakamura M., Tatsumi R., Ikeuchi Y., Mizunoya W. (2015). Data in support of protocol for rat single muscle-fiber isolation and culture. Data Brief.

[25] Komiya Y., Anderson J.E., Akahoshi M., Nakamura M., Tatsumi R., Ikeuchi Y., Mizunoya W. (2015). Protocol for rat single muscle fiber isolation and culture. Anal. Biochem..

[26] Narkar V.A., Downes M., Yu R.T., Embler E., Wang Y.X., Banayo E., Mihaylova M.M., Nelson M.C., Zou Y., Juguilon H. (2008). AMPK and PPARδ agonists are exercise mimetics. Cell.

[27] Richter E.A., Hargreaves M. (2013). Exercise, GLUT4, and skeletal muscle glucose uptake. Physiol. Rev..

[28] Kjøbsted R., Hingst J.R., Fentz J., Foretz M., Sanz M.N., Pehmøller C., Shum M., Marette A., Mounier R., Treebak J.T. (2018). AMPK in skeletal muscle function and metabolism. Faseb. J..

[29] Xie L., Wang D.I. (1996). Energy metabolism and ATP balance in animal cell cultivation using a stoichiometrically based reaction network. Biotechnol. Bioeng..

[30] Blaauw B., Schiaffino S., Reggiani C. (2013). Mechanisms modulating skeletal muscle phenotype. Compr. Physiol..

[31] Liu J., Liang X., Gan Z. (2015). Transcriptional regulatory circuits controlling muscle fiber type switching. Sci. China Life Sci..

[32] Giguère V. (2008). Transcriptional control of energy homeostasis by the estrogen-related receptors. Endocr. Rev..

[33] Fan W., Evans R.M. (2017). Exercise mimetics: impact on health and performance. Cell Metabol..

[34] Gan Z., Rumsey J., Hazen B.C., Lai L., Leone T.C., Vega R.B., Xie H., Conley K.E., Auwerx J., Smith S.R. (2013). Nuclear receptor/microRNA circuitry links muscle fiber type to energy metabolism. J. Clin. Invest..

[35] Chen Z.-P., Stephens T.J., Murthy S., Canny B.J., Hargreaves M., Witters L.A., Kemp B.E., McConell G.K. (2003). Effect of exercise intensity on skeletal muscle AMPK signaling in humans. Diabetes.

[36] Reznick R.M., Shulman G.I. (2006). The role of AMP-activated protein kinase in mitochondrial biogenesis. J. Physiol..

[37] Gan Z., Burkart-Hartman E.M., Han D.H., Finck B., Leone T.C., Smith E.Y., Ayala J.E., Holloszy J., Kelly D.P. (2011). The nuclear receptor PPARβ/δ programs muscle glucose metabolism in cooperation with AMPK and MEF2. Genes Dev..

[38] Manio M.C.C., Inoue K., Fujitani M., Matsumura S., Fushiki T. (2016). Combined pharmacological activation of AMPK and PPARδ potentiates the effects of exercise in trained mice. Phys. Rep..

[39] Ding J., Gou Q., Jia X., Liu Q., Jin J., Shi J., Hou Y. (2021). AMPK phosphorylates PPARδ to mediate its stabilization, inhibit glucose and glutamine uptake and colon tumor growth. J. Biol. Chem..

[40] Shiraki T., Kamiya N., Shiki S., Kodama T.S., Kakizuka A., Jingami H. (2005). Alpha,beta-unsaturated ketone is a core moiety of natural ligands for covalent binding to peroxisome proliferator-activated receptor gamma. J. Biol. Chem..

[41] Waku T., Shiraki T., Oyama T., Fujimoto Y., Maebara K., Kamiya N., Jingami H., Morikawa K. (2009). Structural insight into PPARγ activation through covalent modification with endogenous fatty acids. J. Mol. Biol..

[42] Shearer B.G., Wiethe R.W., Ashe A., Billin A.N., Way J.M., Stanley T.B., Wagner C.D., Xu R.X., Leesnitzer L.M., Merrihew R.V. (2010). Identification and characterization of 4-chloro-N-(2-{[5-trifluoromethyl)-2-pyridyl]sulfonyl}ethyl)benzamide (GSK3787), a selective and irreversible peroxisome proliferator-activated receptor delta (PPARdelta) antagonist. J. Med. Chem..

[43] Xu H.E., Lambert M.H., Montana V.G., Parks D.J., Blanchard S.G., Brown P.J., Sternbach D.D., Lehmann J.M., Wisely G.B., Willson T.M. (1999). Molecular recognition of fatty acids by peroxisome proliferator–activated receptors that activate the PPARs *in vitro* have pharmacological effects similar to those reported for the synthetic PPAR. Mol. Cell.

[44] Liu J., Liang X., Zhou D., Lai L., Xiao L., Liu L., Fu T., Kong Y., Zhou Q., Vega R.B. (2016). Coupling of mitochondrial function and skeletal muscle fiber type by a miR-499/Fnip1/AMPK circuit. EMBO Mol. Med..

[45] Allen D., Westerblad H. (2004). Lactic acid - The latest performance-enhancing drug. Science.

[46] Gladden L.B. (2004). Lactate metabolism: a new paradigm for the third millennium. J. Physiol..

[47] Hui S., Ghergurovich J.M., Morscher R.J., Jang C., Teng X., Lu W., Esparza L.A., Reya T., Zhan L., Yanxiang Guo J. (2017). Glucose feeds the TCA cycle via circulating lactate. Nature.

[48] Hashimoto T., Hussien R., Oommen S., Gohil K., Brooks G.A. (2007). Lactate sensitive transcription factor network in L6 cells: activation of MCT1 and mitochondrial biogenesis. Faseb. J..

[49] Kitaoka Y., Takeda K., Tamura Y., Hatta H. (2016). Lactate administration increases mRNA expression of PGC-1α and UCP3 in mouse skeletal muscle. Appl. Physiol. Nutr. Metabol..

[50] Takahashi K., Kitaoka Y., Matsunaga Y., Hatta H. (2019). Effects of lactate administration on mitochondrial enzyme activity and monocarboxylate transporters in mouse skeletal muscle. Phys. Rep..

[51] Tanida T., Matsuda K.I., Tanaka M. (2020). Novel metabolic system for lactic acid via LRPGC1/ERRγ signaling pathway. Faseb. J..

[52] Gong S., Yin Y., Han M., Guo L., Duan Y., Guo Q., Yin J., Li F. (2023). Dietary leucine and fish oil cooperatively regulate skeletal myofiber type transformation via the CaMKII signaling pathway of pigs. Food Funct..

[53] Arany Z., Lebrasseur N., Morris C., Smith E., Yang W., Ma Y., Chin S., Spiegelman B.M. (2007). The transcriptional coactivator PGC-1β drives the formation of oxidative type IIX fibers in skeletal muscle. Cell Metabol..

[54] Lee J.H., Kishikawa M., Kumazoe M., Yamada K., Tachibana H. (2010). Vitamin A enhances antitumor effect of a green tea polyphenol on melanoma by upregulating the polyphenol sensing molecule 67-kDa laminin receptor. PLoS One.

[55] Yamashita S., Hirashima A., Lin I.C., Bae J., Nakahara K., Murata M., Yamada S., Kumazoe M., Yoshitomi R., Kadomatsu M. (2018). Saturated fatty acid attenuates anti-obesity effect of green tea. Sci. Rep..

[56] Pérez E., Bourguet W., Gronemeyer H., De Lera A.R. (2012). Modulation of RXR function through ligand design. Biochim. Biophys. Acta.

[57] Manickam R., Wahli W. (2017). Roles of peroxisome proliferator-activated receptor β/δ in skeletal muscle physiology. Biochimie.

[58] Yamada H., Oshiro E., Kikuchi S., Hakozaki M., Takahashi H., Kimura K.I. (2014). Hydroxyeicosapentaenoic acids from the Pacific krill show high ligand activities for PPARs. J. Lipid Res..

[59] Sawano S., Komiya Y., Ichitsubo R., Ohkawa Y., Nakamura M., Tatsumi R., Ikeuchi Y., Mizunoya W. (2016). A one-step immunostaining method to visualize rodent muscle fiber type within a single specimen. PLoS One.

[60] Kim D., Langmead B., Salzberg S.L. (2015). HISAT: a fast spliced aligner with low memory requirements. Nat. Methods.

[61] Liao Y., Smyth G.K., Shi W. (2014). FeatureCounts: an efficient general purpose program for assigning sequence reads to genomic features. Bioinformatics.

[62] McCarthy D.J., Chen Y., Smyth G.K. (2012). Differential expression analysis of multifactor RNA-Seq experiments with respect to biological variation. Nucleic Acids Res..

[63] Frayn K.N. (1983). Calculation of substrate oxidation rates *in vivo* from gaseous exchange. J. Appl. Physiol..

[64] Iwata A., Fuchioka S., Hiraoka K., Masuhara M., Kami K. (2010). Characteristics of locomotion, muscle strength, and muscle tissue in regenerating rat skeletal muscles. Muscle Nerve.

[65] Mizunoya W., Miyahara H., Okamoto S., Akahoshi M., Suzuki T., Do M.K.Q., Ohtsubo H., Komiya Y., Lan M., Waga T. (2015). Improvement of endurance based on muscle fiber-type composition by treatment with dietary apple polyphenols in rats. PLoS One.

[66] Nomaguchi K., Tanaka M., Misawa E., Yamada M., Toida T., Iwatsuki K., Goto T., Kawada T. (2011). Aloe vera phytosterols act as ligands for PPAR and improve the expression levels of PPAR target genes in the livers of mice with diet-induced obesity. Obes. Res. Clin. Pract..

[67] Huang D.W., Sherman B.T., Lempicki R.A. (2009). Systematic and integrative analysis of large gene lists using DAVID bioinformatics resources. Nat. Protoc..

[68] Sugimoto M., Wong D.T., Hirayama A., Soga T., Tomita M. (2010). Capillary electrophoresis mass spectrometry-based saliva metabolomics identified oral, breast and pancreatic cancer-specific profiles. Metabolomics.

[69] Yamamoto H., Fujimori T., Sato H., Ishikawa G., Kami K., Ohashi Y. (2014). Statistical hypothesis testing of factor loading in principle component analysis and its application to metabolite set enrichment analysis. BMC Bioinf..

[70] Junker B.H., Klukas C., Schreiber F. (2006). Vanted: a system for advanced data analysis and visualization in the context of biological networks. BMC Bioinf..

