# Supplementary material for: Eicosapentaenoic acid increases proportion of type 1 muscle fibers through PPARδ and AMPK pathways in rats
Source: iScience. 2024 Apr 26;27(6):109816. doi: 10.1016/j.isci.2024.109816 (PMC11108975; doi:10.1016/j.isci.2024.109816)
Supplement: Document S1. Figures S1–S5 and Tables S1 and S2 [file mmc1.pdf]

iScience, Volume *27*

Supplemental information

Eicosapentaenoic acid increases proportion of type 1 muscle fibers through PPARδ and AMPK pathways in rats

Yusuke Komiya, Yuka Sakazaki, Tsuyoshi Goto, Fuminori Kawabata, Takahiro Suzuki, Yusuke Sato, Shoko Sawano, Mako Nakamura, Ryuichi Tatsumi, Yoshihide Ikeuchi, Keizo Arihara, and Wataru Mizunoya

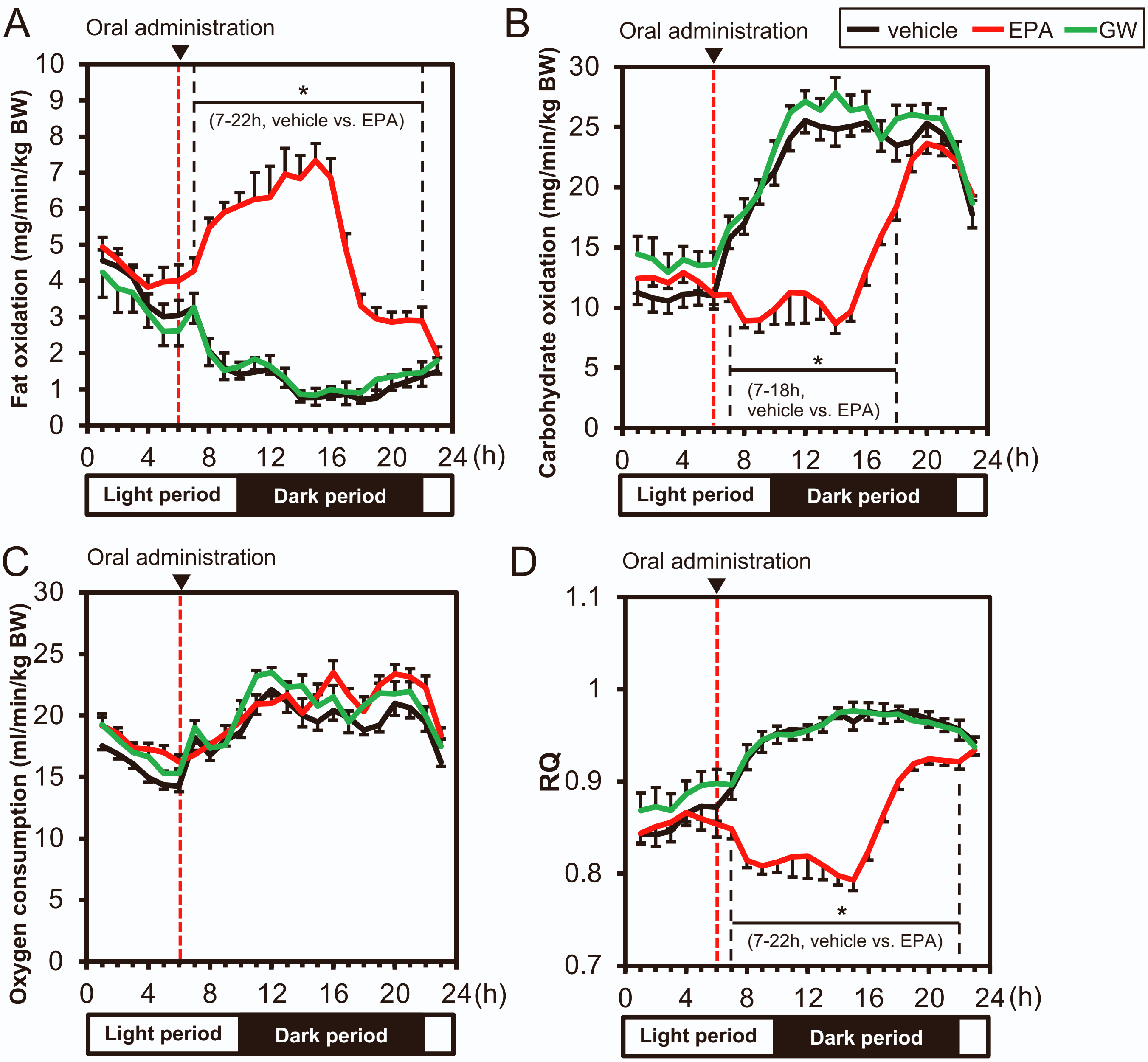

Figure S1. Effect of 7-week EPA supplementation on circadian metabolism of rats, Related to Figure 3.

Time-course changes and calculated fat oxidation (A), carbohydrate oxidation (B), oxygen consumption (C), and respiratory quotient (RQ; D). Free eicosapentaenoic acid (EPA; Biosynth Ltd., Berkshire, UK) was used in the Figure S1 experiment in contrast to EPA ethyl ester used in the main text experiment. Male Fischer F344 rats (6-7 weeks old) were administered with free EPA (4000 mg/kg/day) or GW501516 (5 mg/kg/day) for seven weeks (5 days/week). Gas analysis was performed using an open circuit metabolic gas analysis system connected directly to a mass spectrometer (ARCO-2000; Arco System Inc., Chiba, Japan) for 24 h in the seventh week. During respiratory gas analysis, rats were given water and commercial standard chow ad libitum. Room air was pumped through the chambers at a rate of 0.3 L/min. Expired air was dried in a cotton-thin column and then directed to an O2/CO2 analyzer for mass spectrometry. Rats were administered EPA and GW501516 5 h after the start of the analysis. Fat oxidation was significantly increased, and carbohydrate oxidation and RQ were significantly decreased in the EPA group compared to the vehicle group. However, the increased fat oxidation and decreased RQ could have been induced by the acute oxidation of administered EPA as energy substrates suggested by increased fat oxidation immediately after EPA administration in this pilot study. Data are means ± SEM (n=6, *$P < 0.05$ compared with the vehicle controls).

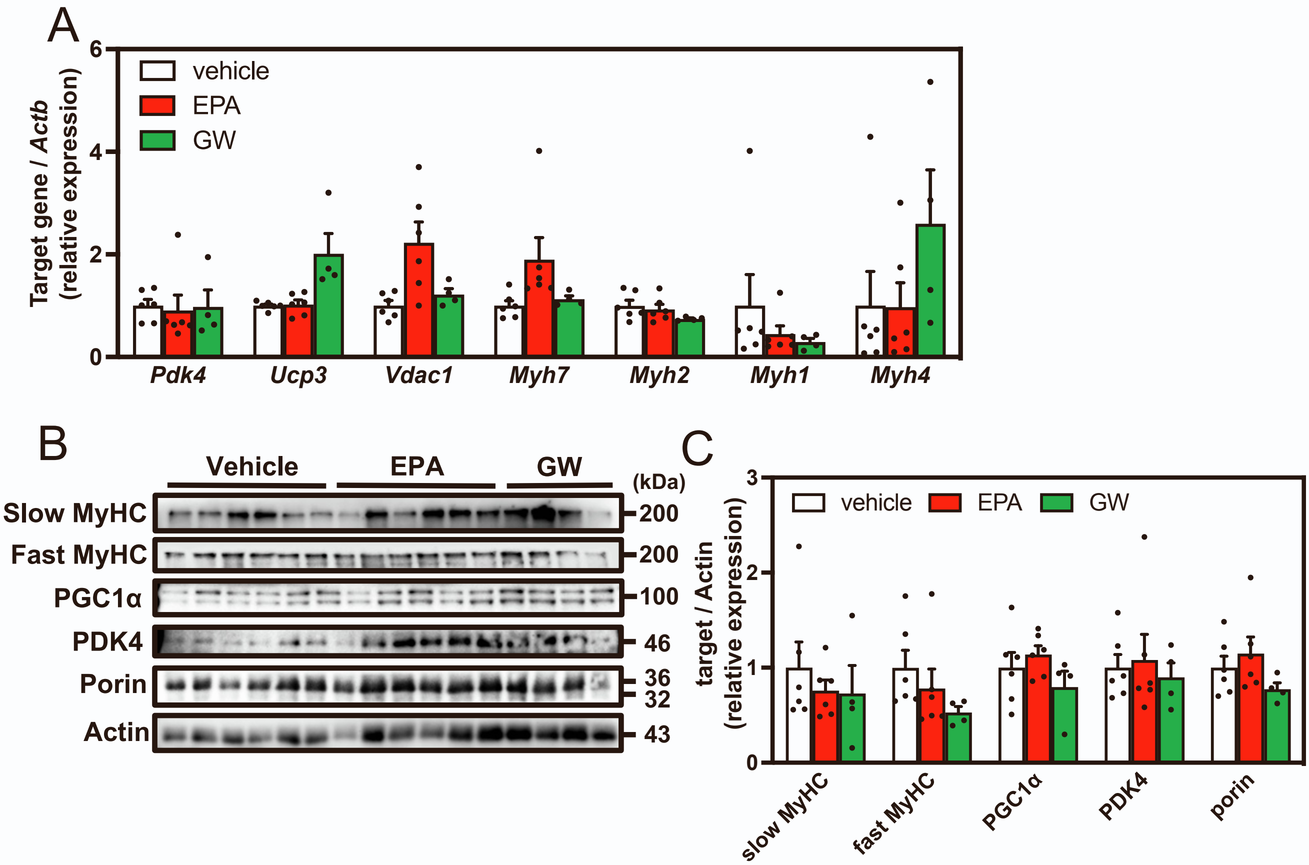

Figure S2. Effects of 4-week EPA supplementation on mRNA and protein expression of muscle fiber type related factors in soleus muscle of rats, Related to Figure 4.
Effects of eicosapentaenoic acid (EPA) supplementation on the mRNA (A) and protein (B and C) expression of muscle fiber type related factors in soleus muscle of rats. The intensity of the immunoblot bands (B) was quantified and normalized to actin as a loading control (C). Data are means ± SEM (n=6 in vehicle and EPA group, n=4 in GW501516 group).

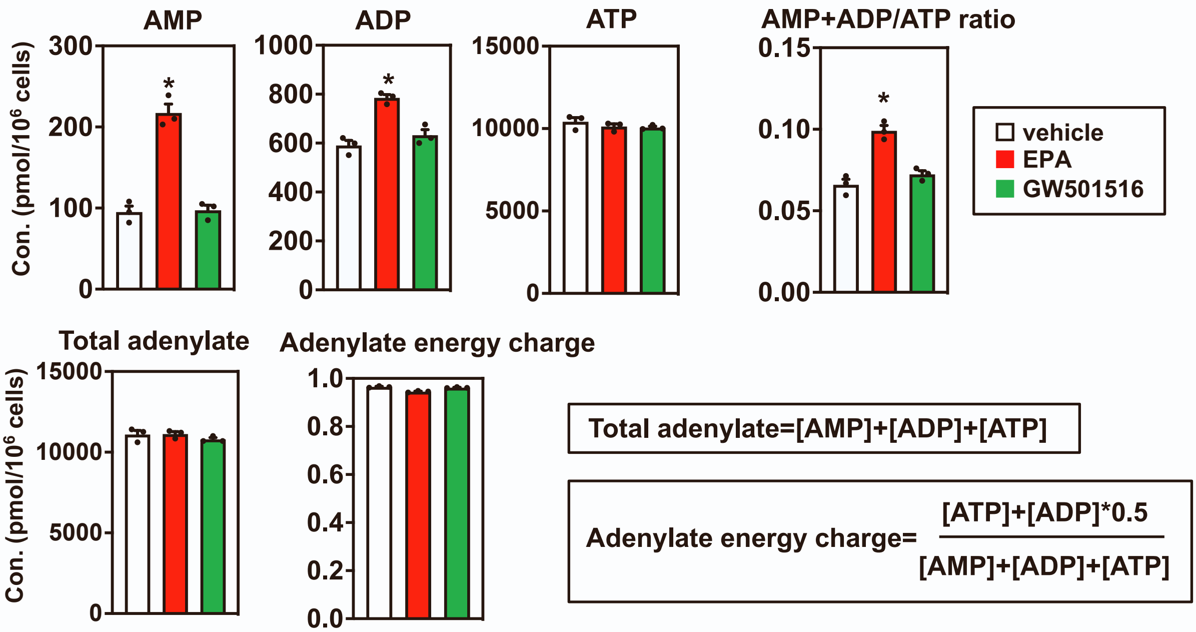

Figure S3. Effects of EPA supplementation on intracellular energy state-related metabolites in L6 myotubes, Related to Figure 6.

Intracellular energy state-related metabolites were quantified in L6 myotubes. Total adenylate and adenylate energy charge were calculated according to the above equations in the squares, respectively. Data are means ± SEM (n=3 independent cultures, *P < 0.05 compared with the vehicle controls).

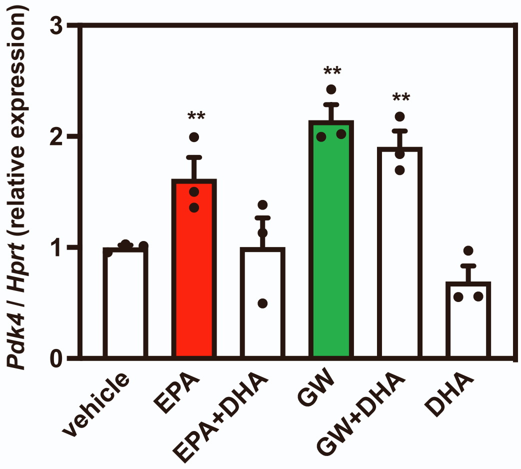

Figure S4. Effect of simultaneous supplementation of EPA and DHA in muscle cells, Related to Figure 1.

Effects of simultaneous supplementation of eicosapentaenoic acid (EPA) and docosahexaenoic acid (DHA) on *Pdk4* expression in muscle fibers isolated from FDB muscle. Cells were treated with each reagent for 12 h. Data are means ± SEM (n=3 independent cultures, **P < 0.01 compared with the vehicle controls).

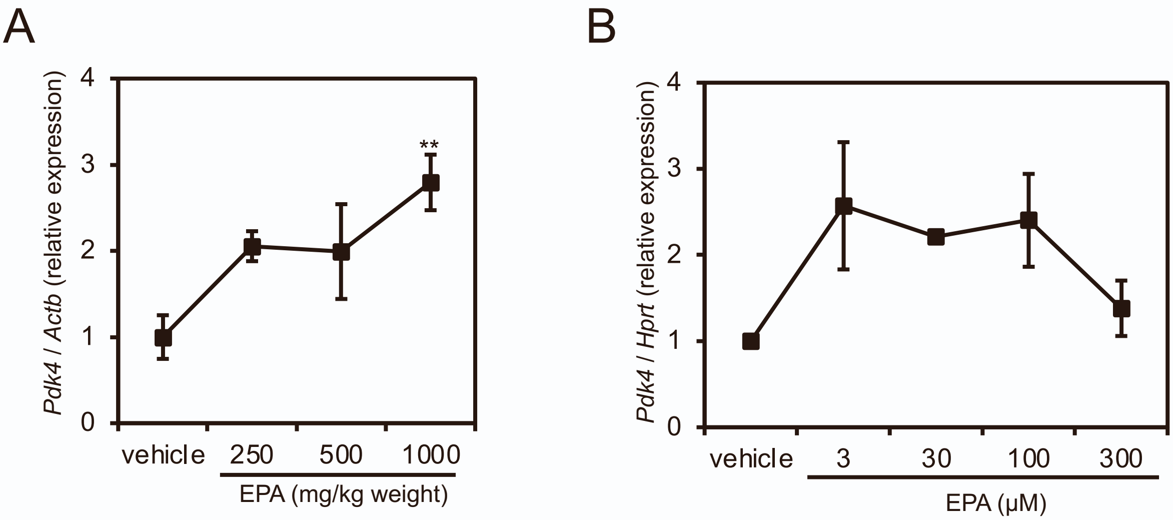

Figure S5. Dose response of EPA in vivo and in vitro, Related to Figure 1-6.
The optimal doses of eicosapentaenoic acid (EPA) were determined by the expression level of *Pdk4* (PPARδ targe gene) in rat extensor digitorum longus (EDL) muscle (A) and cultured muscle fibers isolated from flexor digitorum brevis (FDB) muscle (B). The dose was the amount of orally administered EPA in vivo experiment, and the final concentration of EPA in culture medium in vitro experiment. Data are means ± SEM (n=3 rats or independent cultures, **P < 0.01 compared with the vehicle controls).

Table S1. Growth performance and tissue weights, Related to Figure 3 and 4.

	vehicle			EPA			GW		
Body weight gain and food intake (g)									
Final body weight	201.9	±	2.4	204.4	±	2.7	204.7	±	5.4
Body weight gain	51.9	±	3.6	55.1	±	1.7	51.4	±	6.4
Total food intake	377.7	±	10.6	384.6	±	13.0	407.6	±	9.9
Tissue weight (mg)									
Soleus muscle	98.3	±	14.6	78.7	±	1.3	79.4	±	1.5
Plantaris muscle	203.9	±	4.7	203.7	±	3.3	209.6	±	13.0
Gastrocnemius muscle	1033.0	±	22.5	1040.1	±	19.0	1042.4	±	27.6
EDL muscle	85.6	±	5.8	94.5	±	1.5	102.6	±	4.5
TA muscle	338.1	±	30.4	375.0	±	7.1	381.7	±	11.3
Heart	640.7	±	21.0	648.3	±	5.1	673.3	±	15.0
Liver	6827.8	±	132.3	7444.8	±	372.2	8516.8**	±	311.1
Kidney	1606.1	±	39.8	1668.0	±	37.4	1799.4*	±	43.5
Spleen	465.1	±	12.7	473.4	±	4.2	487.8	±	22.8
Epididymal fat	2336.1	±	152.5	2155.6	±	74.6	2528.0	±	212.0
Perirenal fat	1312.7	±	114.3	1268.8	±	127.2	1742.5	±	300.9
Mesenteric fat	1474.7	±	105.4	1594.0	±	78.5	1750.0	±	228.7
Brown adipose tissue	178.0	±	7.2	173.3	±	4.5	233.5*	±	26.6

Values are means ± SEM (n=6 in vehicle and EPA group, n=4 in GW501516 group, **$P < 0.01$ and *$P < 0.05$ compared with the vehicle control).

Table S2. List of primer sequences for real-time quantitative PCR, Related to Figure 1, 2, and 4.

Gene	Forward (5’-3’)	Reverse (5’-3’)
Angptl4	GGAGGACTTCTCCAAGCCTATT	AGGCCTCTCAGACTTCTGCTT
Cpt1a	GGAGGAAAACACCTGTCCAC	TCAAGGGTGTCCGACCTG
Cs	TGGAGCTGTTATTGGTGCAGT	GGTTCCTTCTTCAAGGACAACTT
Hprt	TTCAAGGCCGTGTTAAGGA	CCTTTGGTGCTAGGCTTGG
Ldha	GTCAAGGCCAAAGCTGTCTC	AACACAAAGTAGGAGCAAAGATGA
Mef2d	GGCGCTACTTCCGAGACTAC	TGGTCAGCAGGTTGTGTGTC
Myh7	GCTGCTGTTGGAAGTCAGC	AGTGTTCGTTCCTCGGAGTG
Myh2	GCGCTGGAGGAGTGTTTTT	CGCTCTCCAGTTGAACCAAG
Myh1	TTTGTGAAATGCCATGACAAG	CAGATGCTTTCTTCTCTTGTTTGT
Myh4	CAAAATGACTCAGGAGGAGTACG	TCCTTGATGTATTGTCTGTCTGG
Pdk4	GTCGGAGAAGCTGGTGGAC	TCAGCTGCCAGGACTGCTA
Ppargc1a	TGACCATCTGCCTTCCAGA	TGTAGGTGGCGCAAGACA
Vdac1	TTCGTGGGTACGTTCTGTGG	TACCAGGGACTGGCATGAGA
Tnni1	GAAAGACAACGGACAAATCACC	GGGGGTGATATGTTTGAACTTG
Ucp3	TGAAAGGGCCAAACAGAGAG	GTAAATCACACGGCGCTCTT

Angptl4, angiopoietin-like4; *Cpt1a*, carnitine palmitoyltransferase 1a; *Cs*, citrate synthase; *Hprt*, hypoxanthine guanine phosphoribosyl transferase; *Ldha*, lactate dehydrogenase A; *Mef2d*, myocyte enhancer factor 2D; *Myh1*, myosin heavy chain 1; *Myh2*, myosin heavy chain 2; *Myh4*, myosin heavy chain 4; *Myh7*, myosin heavy chain 7; *Pdk4*, pyruvate dehydrogenase kinase 4; *Ppargc1a*, peroxisome proliferative activated receptor, gamma, coactivator 1 alpha; *Vdac1*, voltage-dependent anion channel 1; *Tnni1*, troponin I; *Ucp3*, uncoupling protein 3.
